# Help-seeking attitudes and behaviours among youth with eating disorders: a scoping review

**DOI:** 10.1186/s40337-022-00543-8

**Published:** 2022-02-14

**Authors:** Maria Nicula, Danielle Pellegrini, Laura Grennan, Neera Bhatnagar, Gail McVey, Jennifer Couturier

**Affiliations:** 1 McMaster University, 1200 Main Street West, Hamilton, ON L8N 3Z5 Canada; 2 University Health Network, Toronto, ON Canada; 3 University of Toronto, Toronto, ON Canada; 4 McMaster Children’s Hospital, Hamilton, ON Canada

**Keywords:** Help-seeking, Treatment-seeking, Mental health, Eating disorders, Youth, Children, Adolescents, Emerging adults, Early intervention, Health promotion

## Abstract

**Background:**

Although early intervention is crucial in interrupting the development of eating disorders, little is currently known about help-seeking behaviours among individuals experiencing eating disorder symptoms. Given that eating disorders typically begin early in life, it is necessary to investigate the processes employed by children, adolescents, and emerging adults when seeking services for troubling symptoms. This is a growing concern as the COVID-19 pandemic has resulted in an increase in the number of individuals engaging in disordered eating behaviours. This scoping review explores the current state of the literature for evidence on how youth with eating disorder symptoms seek help, with the aim of better understanding how to identify and treat more individuals earlier.

**Methods:**

Using scoping review methodology, we searched seven databases for studies published from January 2000 to April 2021 that reported on help-seeking attitudes, behaviours, and healthcare utilization patterns for children and adolescents (< 18 years), emerging adults (18–25 years), and a mixture of these groups (< 25 years). Seven thousand, two hundred, and eighteen articles were identified for review. After duplicates were removed, three reviewers independently screened titles and abstracts and reviewed full-text articles. Findings related to help-seeking activity were extracted from the 62 articles that were ultimately included in this scoping review.

**Results:**

Study findings were summarized into help-seeking patterns (i.e., rates, types) as well as factors ranging from the individual level to society that influenced help-seeking behaviour. Many youth meeting eating disorder criteria were not seeking help. Notable barriers to help-seeking included poor mental health literacy, experiences with healthcare providers who failed to detect and lacked knowledge about eating disorders, minimal support from family and friends, and stigma surrounding eating disorders and help-seeking for mental health concerns.

**Conclusions:**

The results of this scoping review can be used to inform early intervention and health promotion program development. Future research should focus on the help-seeking attitudes and activities among underrepresented groups with eating disorders (e.g., men, ethnic and gender minorities), the perspectives of family and other supporters in the help-seeking process for youth, and retrospective accounts from adults with lived experience of an eating disorder.

**Plain English summary**

Addressing and interrupting eating disorder-related thoughts and behaviours as soon as possible, with the help of a mental health professional, leads to better outcomes for youth struggling with an eating disorder. However, little is known about what prompts youth to talk about their weight, body, or eating concerns with someone—like their parent, friend, teacher, guidance counsellor, or primary care practitioner. This review explores the available published research on help-seeking patterns and preferences among youth with eating disorder concerns. Our team followed a standardized process to find 62 relevant articles for this paper. Of note, many young people who reported eating disorder concerns were not seeking help for themselves. Feeling supported by family and their primary care provider, understanding the signs of an eating disorder, and not feeling shame for reaching out for help reportedly led youth to speak up about their concerns. The findings have clinical implications for learning effective ways to help youth feel safe to speak freely about their eating disorder-related concerns, which enhances the chances of intervening early and catching symptoms before they worsen.

**Supplementary Information:**

The online version contains supplementary material available at 10.1186/s40337-022-00543-8.

## Background

Stigma surrounding mental illness remains highly prevalent in today’s society and, as a result, interferes with the ability of those struggling with mental health concerns to seek help for their issues [1]. While difficult, seeking help for one’s mental illness early is highly beneficial as it has been associated with better and faster recovery, effective symptom management, and improved quality of life [2–4].

Help-seeking behaviour is influenced in part by the age of the individual; youth are less likely to seek help than adults due to stigmatizing or negative attitudes towards seeking professional help, a lack of mental health literacy, and a general belief that one should be able to recover independently of treatment [5, 6]. Sex and gender also impact help-seeking behaviours; for example, women are more likely than men to seek professional help [6, 7]. Ethnic minorities, individuals with low income, and other underrepresented groups are more likely than their counterparts to face systemic healthcare barriers throughout the process of seeking professional help for one’s mental illness [8–10]; some examples of this include culturally-specific stigma against seeking formal help, not having the resources necessary to access help, or healthcare providers’ inability to detect the disorder. The frequency and characteristics of help-seeking behaviours are also related to the type of mental health concern. For instance, individuals who suffer from mood or anxiety disorders are more likely to seek help compared to individuals who suffer from substance abuse or eating disorders [11].

Eating disorders (EDs) represent a class of psychiatric disorders that benefit significantly from earlier recognition and treatment [12]. Specifically, recovery from an ED becomes much less likely the longer the illness has persisted prior to seeking treatment [13]. Even so, little is known about the help-seeking behaviours among youth experiencing signs and symptoms of EDs. These disorders negatively impact the mental and physical health of those who struggle with the illness, and severely impact the well-being of their families [14]. If left untreated, individuals with EDs are at an increased risk of developing serious physical, psychological, and social difficulties and, in extreme cases, death [15].

There is evidence to suggest that help-seeking behaviour is strongly associated with one’s mental health literacy [7, 16, 17]. The term *mental health literacy* (MHL), coined by Jorm and his colleagues in 1997 [18], involves various components such as, but not limited to, recognizing developing signs of a disorder, awareness of help-seeking options and treatments available, and knowledge of effective self-help strategies for milder problems [19]. Ultimately, MHL “is knowledge that is linked to the possibility of action to benefit one’s own mental health or that of others” [19]. In response to the association between MHL and help-seeking behaviour, researchers have suggested that health promotion programs that build MHL can help to increase help-seeking behaviour. Evidence suggests that this would address commonly held beliefs about how these disorders present or alleviate doubts about treatment among individuals with common mental disorders and others in their vicinity, such as their mental health care providers [7, 16, 20]. Although the mental health field has experienced increased interest in health promotion and early intervention approaches in more recent years [17, 21], there is currently a lack of—and need for—evidence-based research that explores the effectiveness of these programs when educating various stakeholder groups [20, 22, 23].

During the COVID-19 pandemic, the number of individuals diagnosed with an ED and the number of adolescents engaging in disordered eating behaviours and attitudes have increased dramatically [24]. Social media promotes messaging about “undesirable” and “inevitable” weight gain related to lockdown restrictions, promoting shape and weight concerns, and restrictive and compensatory behaviours among those with, or at-risk for, EDs [25–28].

Given that health promotion and early intervention initiatives have the potential to motivate youth to seek help for their ED symptoms, the objective of this scoping review is to explore the available evidence on help-seeking rates, preferred sources of help, and factors that influence help-seeking behaviours from the perspectives of children, adolescents, and emerging adults with, or who meet the criteria for, an ED. The findings from this scoping review are intended to shed light on patterns of help-seeking and healthcare utilization prior to the development of full syndrome EDs to inform the creation and planning of health promotion and early intervention programs—both of which have been neglected in the literature compared to selective prevention and clinical management approaches [20, 22].

## Methods

We used scoping review methodology [29–32] to ensure that we collated a variety of evidence on help-seeking behaviours and attitudes as well as healthcare utilization for children, adolescents, and emerging adults experiencing ED symptoms. To structure the literature search strategy for this review, we followed the five stages outlined in the Arksey and O’Malley framework [29].

### Stage 1: Identifying research questions

Two questions guided this scoping review. Firstly, how often, when, and where do children, adolescents, and emerging adults attempt to receive help when concerns or early warning signs of disordered eating first appear? Secondly, what are the factors that influence children, adolescents, and emerging adults to seek help for their disordered eating?

### Stage 2: Identifying relevant studies

#### Eligibility criteria

We included all research literature written in any language, including quantitative, qualitative, and mixed methods papers, on help-seeking for children and adolescents (< 18 years) and emerging adults (18–25 years) regarding disordered eating. During the screening process, the citation reviewers agreed to include studies whose participants had a mean or median age of up to and including 25 years. We excluded studies primarily involving adults (mean or median age of > 25 years), unless the adults were speaking about their help-seeking experiences as youth (≤ 25 years). Additionally, studies that mentioned treatment-seeking samples but did not contain relevant information pertaining to participants’ attitudes, behaviours, or utilization patterns for seeking help were also excluded from the review.

#### Databases and literature search strategy

We conducted a systematic search using seven databases: OVID Medline, PsycINFO, EMBASE, Cochrane Database of Systematic Reviews, CENTRAL, EMCARE, and CINAHL. The search included articles from January 2000 to April 2021, to ensure that timely evidence was included. The searches included a combination of keywords and subject headings for each concept. The sample search strategy included, but was not limited to, various combinations of the following terms as appropriate for the research questions: disordered eating OR body dissatisfaction OR eating concerns OR shape concerns OR weight concerns OR dietary restraint AND help-seeking attitudes OR help-seeking behaviours OR help-seeking OR early intervention OR healthcare access OR treatment seeking OR motivation to seek treatment. The references of relevant articles obtained were also reviewed. We followed the definition of help-seeking as defined by The World Health Organization (WHO), which is “any action or activity carried out by an individual who perceives a need for personal, psychological, or affective assistance, with the purposes of meeting this need in a positive way. The ‘help’ provided might consist of a service (e.g., medical consultation, clinical care, medical treatment, or a counselling session), a referral for service, or for follow-up care, and delivered by clinic services, counsellors, psychologists, medical staff, youth programmes, peer groups, friends, and family members” [33].

### Stage 3: Study selection

Two authors independently screened the results generated by the searches and came to a consensus on which studies met eligibility criteria. Duplicate records were removed. DistillerSR was used for article screening and data extraction and Endnote was used to organize the studies. These two reviewers excluded reports with titles and abstracts that were irrelevant to the review. Potentially relevant articles were reviewed in full text by the two reviewers who had to agree on their inclusion. If agreement on abstract or full article inclusion could not be reached between the two reviewers, an opinion was requested from a third reviewer. However, there were no disputes.

### Stage 4: Data charting process

A data-charting electronic form on DistillerSR was jointly developed by the two reviewers to determine which variables to extract. Two authors independently extracted data, while updating the data-charting form as needed. Data was extracted on the following items: general data (title, year of publication, author’s name, country), type of paper (e.g., qualitative, cross-sectional, mixed methods), age range, sample size and description of sample, methodology, outcomes, type of analysis, and results related to help-seeking. In line with standard scoping review practice and methodology, we did not perform a formal critical appraisal of primary studies [34, 35].

### Step 5: Summarizing results

The research questions guiding the search strategy led to the creation of distinct result categories. Help-seeking rates and preferred and actual sources of help represent the categories that emerged from the data addressing the first research question, while facilitators and barriers to help-seeking addressed the second research question. The findings from papers that included samples with a median or mean age younger than 18 years of age were labeled as “children/adolescents”, studies involving samples with a median or mean age between 18 and 25 years of age were labelled as “emerging adults”, and studies that contained individuals from both of these age groups (e.g., age ranges overlapped or did not report a median or mean age) were labeled as “mixed population”. The terms “help-seeking” and “treatment-seeking” were used interchangeably. We reported the review following the Preferred Reporting Items for Systematic Review and Meta-Analysis (PRISMA) guidelines—extension for scoping review [36].

## Results

**Fig. 1:**
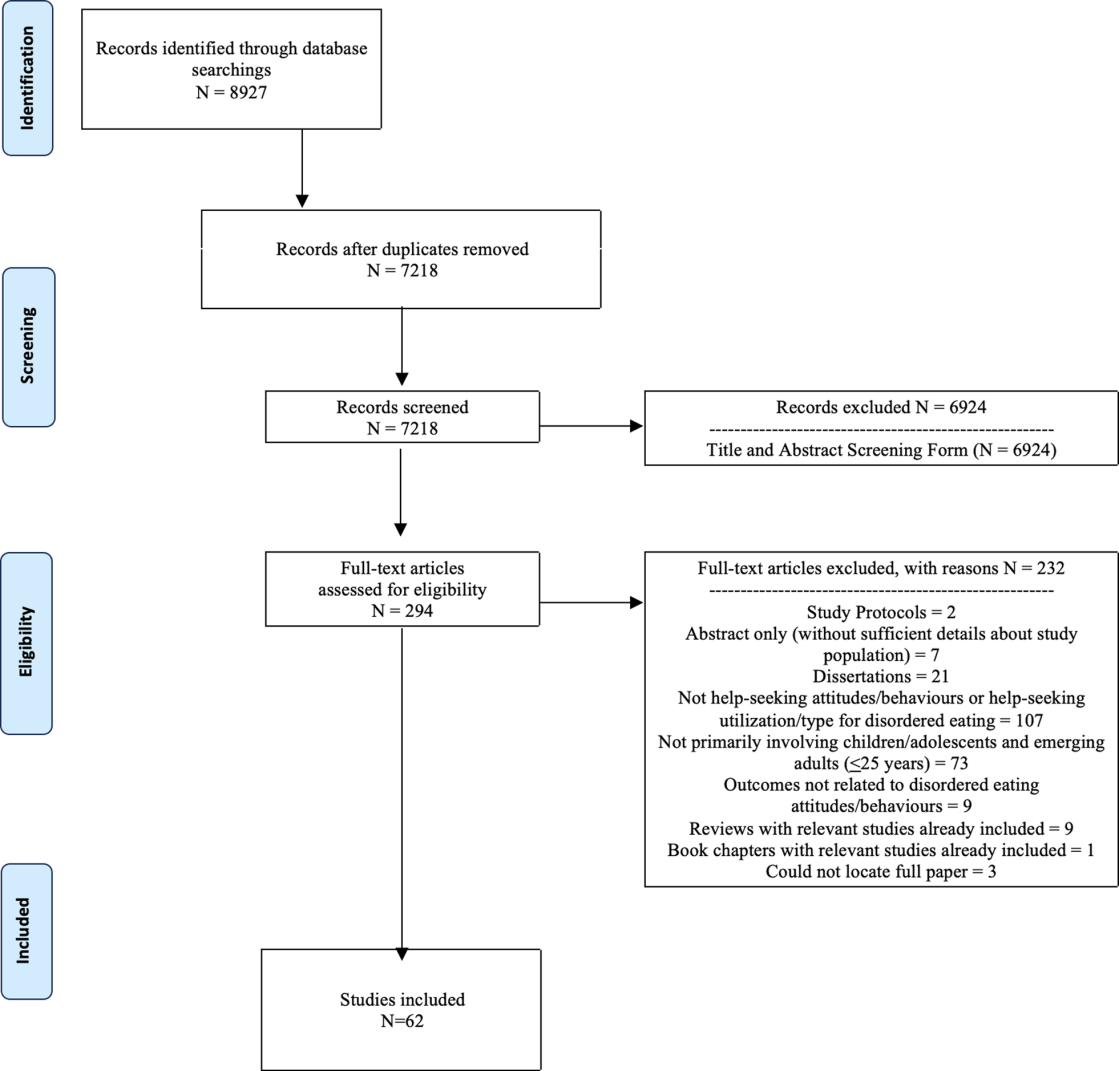
PRISMA flow diagram for this scoping review’s literature search process

Seven thousand, two hundred and eighteen abstracts were identified for review (see PRISMA flow diagram in Fig. 1). After duplicates were removed, abstracts screened, and full-text articles reviewed, the results from 62 papers were included in this section. No additional abstracts were identified through the review of reference lists. See Additional file 1 to view all of the extracted data from this scoping review, organized by age group.

### Help-seeking rates

#### Children/adolescents

Six cross-sectional studies reported help-seeking rates among children and adolescents. Thirty-four percent of an Irish high-school student sample (n = 290) reported being concerned about their eating habits; of these, approximately half had discussed these concerns with someone else [37]. More than 85% of an Australian adolescent sample (n = 922), who experienced disordered eating in the past year, reported help-seeking—typically via self-help [38]. Only 1.7% of 116 middle and high-school students meeting ED criteria reported being in therapy [39]. Ten percent of an Australian (n = 1002) and 20% of an American (n = 281) adolescent sample that met ED criteria reported seeking treatment for their body image problems and/or ED [40–42].

#### Emerging adults

Five cross-sectional studies, one longitudinal study, and one mixed methods study explored the willingness to seek help among emerging adults with eating concerns. To start, 61% of 74 university-age women meeting subclinical ED criteria and 44% of 32 meeting clinical ED criteria did not believe they needed counselling [43]. Similarly, 48% of 205 university students who screened positive for an ED perceived a need for help [44]. Of the 109 college students who were recommended for further clinical evaluation after participating in the National Eating Disorders Screening Program (NEDSP), 51% were willing to follow up with this recommendation, 42% were neutral or had little interest, and 8% were unwilling to do so [45]. Around 50 to 60% of 291 emerging adults from Australia with concerns about their eating, weight, and body shape reported that they would seek help from a mental health professional or a family member [46]. In a longitudinal study, only 21% of 136 university students with high levels of eating concerns (as determined by their Eating Attitudes Test [EAT-26] score) who attended psychoeducational programming about ED awareness stated that they would seek help for themselves [47]. Conversely, 77% of 185 individuals in a mixed methods study who either self-diagnosed as having Orthorexia Nervosa (ON) or posted ON-related content on social media reported that they considered treatment at some point [48]. Lastly, 82.6% of 172 individuals experiencing ED symptoms who visited the Reach Out and Recover Website—an online resource for ED support—indicated that they planned to get treatment [49].

Two cross-sectional studies explored help-seeking rates among adults aged 18 and older from general respondent samples. Thirty-five percent of 102 American university-age women communicated their eating and body image concerns to their primary care providers [50]. Similarly, more than one third of 291 Australian emerging adults with concerns about their eating, weight, or body shape reported having previously sought help for these concerns [46].

One mixed methods study, four cross-sectional studies, and one qualitative study found various rates of help-seeking activity among emerging adults who meet ED criteria. In a mixed methods study, 19 South Australian women who met ED criteria based on the Eating Disorder Examination-Questionnaire (EDE-Q) and had not received a formal diagnosis or any treatment for disordered eating were recruited [51]; due to the sampling criteria for this study, none of these women sought professional help for their disordered eating. Similarly, no participants from a sample of 122 female college women who met criteria for eating concerns reported seeking treatment from a medical professional, while about one fourth reported seeking assistance from adjunct services, such as a nutritionist or a support group [52]. Thirty-eight percent of 130 college-age females who participated in an ED intervention program reported having tried individual counselling for eating concerns at least once, and 11% reported having been hospitalized for their ED prior to college [53]. Sixty-five percent of 43 young Latina women with a history of Binge Eating Disorder (BED) or Bulimia Nervosa (BN) reported seeking treatment for their disorder [54]. Ninety-four percent of 16 female university athletes who recovered from an ED reported receiving professional help [55]. Lastly, among 622 adults living with a lifetime ED who responded to a national epidemiological survey, help-seeking rates were found to differ depending on ED type; 34.5%, 62.6%, and 49.0% of individuals with Anorexia Nervosa (AN), BN, and BED respectively sought help for their ED [56].

Two cross-sectional studies and one open trial reported on similar rates from efforts designed to motivate participants to increase help-seeking activity. Firstly, out of 647 American university students who screened positive for significant ED symptoms or elevated weight concerns, 86.5% had not yet received treatment [57]. Participants in this study were referred to either universal prevention or tailored intervention programs based on the severity of their ED symptoms. Program enrolment was found to be highest among the 276 participants who were assigned a tailored intervention (18.1%) and lowest for the 1163 students assigned to receive universal prevention programming (4.1%). In an open trial studying 61 American university students who screened positive for AN and were provided treatment referral through the Healthy Body Program, 33% reported already being in treatment at the time of referral, 26% had since initiated treatment, and 41% did not initiate treatment [58]. Lastly, only 13% of 121 university female students agreed to be contacted regarding participation for ED prevention studies [59].

#### Mixed population

Two cross-sectional studies involving mixed populations of children, adolescents, and emerging adults discussed intention to seek help. Among 16,396 respondents who screened positive or at risk for an ED using the National Eating Disorders Association (NEDA) screening tool, 10.3% reported they would definitely seek help, 23.4% probably, 40.8% probably not, and 25.5% would not; at two-month follow-up, 15.5% subsequently initiated treatment while 75.5% did not initiate or were not already in treatment [60]. In a different sample who completed NEDA’s screening tool during National Eating Disorders Awareness Week and met criteria for a subclinical or clinical ED (n = 20,727), around 75% reported no intention to seek treatment [61].

Four cross-sectional studies with mixed-age samples reported on the help-seeking history of those who presented with some level of ED symptomology. Only 14% of 405 youth who engaged with pro-ED social media had received treatment, despite their high level of ED symptomatology [62]. Emergency department patients at an American medical centre who screened positive for an ED (n = 1920) were 1.6 times more likely to have previously visited the emergency department than patients who screened negative for EDs [63]. Out of 317 individuals with a diagnosed ED, 57.1% were currently receiving treatment for their ED, 32.8% previously received treatment, and 7.3% never received treatment [64]. The National Eating Disorder Information Centre (NEDIC) reported a notable increase in people seeking help and resources for ED-related symptoms during the early months of the COVID-19 pandemic in 2020 (n = 609) compared to the corresponding time period in previous years, such as in 2018 (n = 394) [65].

In an open trial studying help-seeking rates among 453 at-risk youth participating in ProYouth, an online program for prevention and early intervention for EDs, 9.5% started treatment after three months of using the program, 7.8% intended to start treatment but had not yet, and 43.1% reported that they would seek professional help if they had a need for it [66]. Taken together, these findings indicate that children, adolescents, and emerging adults experiencing ED symptoms range widely in their interest levels, self-reported intentions, and previous experiences seeking help.

### Preferred and actual sources of help

#### Children/adolescents

Five cross-sectional and two qualitative studies with child and adolescent samples discussed preferred and actual sources of help among those with and without ED concerns. Five hundred and eleven British secondary school students were asked how they would prefer to communicate their ED concerns, if they had any, to a teacher [67]. Seventy-three percent preferred to discuss these issues face to face, 22% preferred to write their concerns or send an e-mail, and 5% preferred to send a text message to them. Generally, these students perceived support from non-teaching school staff, such as counsellors, to be helpful. Out of 51 high school students with eating concerns in Ireland, 59% reached out to family for help with their problem, 33% from a friend, and 8% reached out to a health professional or support organization [37]. Among 85% of 922 adolescents from Australia with disordered eating who reported seeking help in the past year, most opted for self-help methods while less than half sought treatment from school-based, primary care, or specialist services [38]. One qualitative study found that many children and adolescents struggling with an ED used an online forum for support, and those who used it found that it offered a trusting and friendly environment, allowed them to be anonymous, and was always available, unlike crisis lines or group support [68]. Speaking virtually with strangers with lived experience was helpful and motivating for newcomers to the forum to seek out help. The majority of a pediatric sample receiving inpatient treatment for AN (n = 258) reported having seen a pediatrician, child/adolescent psychiatrist, or general practitioner (GP) prior to specialized ED treatment [69]. A group of 101 Australian school students meeting ED criteria, all of whom sought help for their body image concerns, reported seeking this help from GPs or doctors, with less receiving specialist care from psychiatrists or therapists [41]. Ten qualitative study participants who received AN treatment as a teenager were often first seen by community practitioners who, in their opinion, lacked experience in EDs [70].

#### Emerging adults

Three cross-sectional studies reported on preferred sources of help and six cross-sectional studies and one qualitative study reported on actual sources of help sought by general and ED samples. When presented with a vignette about a woman expressing symptoms of BN, 255 university-age female students from Singapore identified the following interventions as most helpful to treat her problem: consulting a primary care practitioner, counsellor, or psychologist, seeking advice from a female family member or friend, getting diet or nutrition advice, and taking vitamins and minerals [71]. Participants in this study were less positive about seeing a psychiatrist and were ambivalent about psychotropic medication use. Among 99 female university students who met ED criteria, participants reported that they would go to their friend first and a dietician second if they had weight or eating concerns [72]. They also reported that they would prefer ED support in the form of individual therapy first and group therapy second, and that they would pick a close friend as the person to support their work in therapy. Among 291 emerging adults with eating-related concerns, 60% reported that they would seek help from a mental health professional, 60% said their partner or friend, and 50% indicated that they would seek help from their family doctor, websites, or their parents [46].

In hindsight, 35 American university students who first sought help for their eating and body image concerns from their GP reported wanting to disclose these concerns to friends, family, and specialized mental health providers instead [50]. Twenty percent of respondents in a cross-sectional study reported seeking help from a mental health professional, while 13 to 15% sought help from their GP, parents, websites, friends, or partner [46]. Among 28 young Latina women who sought treatment for BED or BN, 68% received treatment from a psychologist or therapist for individual therapy or from a nutritionist or dietician [54]. Members of this sample endorsed therapist- or psychologist-led group therapy and other treatment providers (i.e., “specialist on EDs”, social worker, yoga teacher, endocrinologist) as being the most helpful, while physicians were rated as least helpful. Friends and family were reported as the main sources for help-seeking in emotionally stressful scenarios among 370 French university students who screened positive for an ED [73]. Specifically, those with restrictive EDs sought help from a psychologist or a specialist practitioner during high-stress situations. Similarly, 37.5% of 20 Spanish individuals receiving outpatient treatment for AN first disclosed their ED symptoms with relatives or partners, while 37.5% shared them with friends [11]. Sixteen female university-age athletes who had recovered from an ED reported nutritionists and social workers as being helpful in their recovery [55]. The most frequently used forms of help reported by 622 American adults with a lifetime ED were counselors, therapists, doctors, and psychologists, followed by the use of self-help or a support group [56]. Lastly, emerging adults reported seeking treatment based on their screening results from online screening tools, such as NEDSP and NEDA, recommending that they pursue further clinical ED evaluation [45, 58]. Similarly, an 18-year-old female experiencing ED symptoms featured in a case report credited ProYouth—specifically the chat session feature—as contributing to her decision to seek psychotherapy [74].

#### Mixed population

One qualitative and three cross-sectional studies explored the preferred sources of help among a mixed population of children, adolescents, and emerging adults. Youth samples meeting ED criteria across two studies reported preferring to seek help from mental health professionals or medical doctors [61, 62]. Eighty-four percent of one of these studies’ samples (n = 405) also reported an interest in trying a mobile mental health program with a human component [62]. These preferences were mirrored in another cross-sectional study where 67.4% of 694 individuals made an appointment with a mental health professional and 47% did the same with a medical doctor after screening positive for an ED [60]. Engagement with online programs for prevention and early intervention of EDs, such as ProYouth and the Proud2BMe website, motivated individuals from two other studies with ED concerns and symptoms to seek treatment [66, 75]. Lastly, a qualitative sample of 10 British men with an ED reported that their first contact with formal services was usually with their GP and their experiences with professionals ranged from positive (e.g., given rapid referral, willingness to offer support) to negative (e.g., delayed referrals, misdiagnosis) [76].

### Facilitators and barriers to help-seeking

Various facilitators and barriers to help-seeking were identified in the scoping review. These factors were organized based on their proximity to the individual experiencing ED symptoms—starting at the individual, familial, and systemic levels and ending with the societal level.

#### Individual

Factors that influenced the decision to seek help among children, adolescents, and emerging adults experiencing ED symptoms were identified in 45 of the included studies.

##### Demographics

**Children/adolescents** Three cross-sectional studies found that children and adolescents who were female, older, sexual minorities, held emigrant status, and had at least one parent graduate from college or university were more likely to seek help [38, 41, 42].

**Emerging adults** In one longitudinal and two cross-sectional studies with emerging adult samples, it was similarly found that males and ethnic or racial minorities were less likely to seek help for their ED, were more likely to hold less positive help-seeking attitudes, and reported seeking help significantly later, than their female or non-ethnic minority (e.g., non-Hispanic Whites, European Americans) counterparts [56, 77, 78].

**Mixed population** Two studies with a mixed population sample found that female, gender minority, and older individuals were more likely to report their intentions to seek help than their younger or male counterparts [60, 66]. One retrospective cross-sectional German study found that individuals with a late onset of AN (age 19 and older) were more likely to be internally motivated (68.1%) to seek help than the intermediate onset group (ages 14–18; 50.9%) and early onset group (ages 14 and younger; 37.5%) [79].

##### ED characteristics

**Children/adolescents** Two cross-sectional school-age samples with a major ED (i.e., AN, BN) were more likely to seek treatment than those with an unspecified or other specified ED [41, 42]. In an American study that explored how ED symptom severity influenced help-seeking attitudes among high school students, the symptomatic group (i.e., exhibiting some ED symptoms) and ED group (i.e., meeting ED criteria) both lacked interest in receiving help, but each group offered a different reason for avoiding counselling [39]; the symptomatic group was more likely to deny their need for counselling, while the ED group was more reluctant to share their problems with others. Additionally, reduced functioning in other areas of life was reported to motivate school students meeting ED criteria from two cross-sectional samples to seek help or use treatment services [38, 41].

**Emerging adults** Characteristics related to one’s ED were found to facilitate or hinder help-seeking among populations of emerging adults. In two cross-sectional studies, young adults were more likely to seek help when their ED symptoms were elevated—specifically symptoms related to AN, BN, and BED [46, 49]. Another factor that positively influenced help-seeking behaviour among emerging adults was when other aspects of life suffered because of the ED [11], such as the inability to compete in sport [55], when their close relationships were affected [48], and when experiencing emotional distress and health concerns [58]. On the other hand, greater levels of disordered eating cognitions (e.g., fear of gaining weight), psychological distress and inflexibility, and pleasantness of ED symptoms in comparable samples were conversely found to be associated with less favourable help-seeking attitudes [11, 78, 80].

Gender was reported to interact with ED characteristics to influence help-seeking attitudes and behaviours. Among women who showed lower restraint levels and men who showed higher restraint in a sample of 3291 American university students, increased ED stigma and increased negative affect were associated with an increased perceived need for treatment [81]. Additionally, among 145 university-age students with disordered eating, help-seeking stigma acted as a stronger barrier for men than for women and one’s anticipated benefits regarding seeking counselling was more likely to facilitate women than men to seek help [82].

**Mixed population** Experiencing higher levels or a greater severity of symptoms motivated members from the following mixed-age samples to seek help: female AN patients new to ED treatment [83], youth receiving outpatient treatment for AN or BN [84], individuals experiencing ED symptoms and their caregivers who contacted NEDIC [65], and individuals engaging with the Proud2Bme website [75] and the ProYouth program [66].

##### Attitudes, beliefs, and fears

**Children/adolescents** Five studies found various attitudes held by children and adolescents with EDs to influence help-seeking activity. Adolescents admitting to having ED symptoms and related impairment were more likely to seek ED treatment [42]. Among students meeting ED criteria, those who recognized they had a body image problem were 2.71 times more likely to seek help than those who did not [40]. The perception that their behaviours were not problematic enough to merit counseling, denying that they have a problem, fear of disclosing their problems to others, not wanting anyone to know, and not knowing who to tell also negatively impacted help-seeking behaviours among two similar populations [37, 39]. In a retrospective qualitative study, female university students (n = 14) with a current or previous ED reported that a ‘snapping’ moment jolted them out of denial in order to get help [85]. This study also found that having a high need for control negatively affected help-seeking attitudes.

**Emerging adults** Multiple studies reported that emerging adults with eating concerns and subclinical or clinical EDs wanted to deal with their illness alone, without seeking help [11, 45, 46, 57, 58, 73, 86]. Similarly, the desire to shield their family from the pain that treatment would cause hindered emerging adults with EDs (n = 34) from seeking social support for their ED [87]. A group of 19 women meeting ED criteria did not seek help for their symptoms as they felt recovery was not possible [51]. This was mirrored by the finding that less than 30% of university female students from Singapore believed treatment would result in full recovery for the individual with BN presented to them in a vignette [71]. Additionally, in a study involving university-age students with EDs, not perceiving a need for help served as a help-seeking barrier [44, 45, 57, 58, 86–88]. Many participants minimized or denied the severity or presence of the problem [11, 46, 51, 87, 89]. or hoped that symptoms would disappear [11, 44]. In fact, women with disordered eating not in treatment felt that their symptoms provided them with a sense of safety [51] or that the benefits brought upon by their symptoms outweighed the costs of seeking help [86]. Multiple fears hindered emerging adults with eating concerns or EDs from seeking help, including a fear of weight gain [46, 51, 86], rejection [90], and doctors [73]. The desire to keep their disorder and habits hidden was reported by six emerging adults in ED recovery as an influence on their ambivalence to reach out for help [91].

In terms of facilitating help-seeking attitudes, comments from others that one’s ED symptoms are dangerous motivated individuals with BN (n = 44) with traits of high efficacy—the belief that they can seek and receive help—but not those with traits of low efficacy [92]. Among 200 adults seeking ED support, intention to seek treatment was positively associated with motivation and confidence to achieve change and a greater recognition of the impact the ED had on relationships and well-being [49]. Fearing an increase in symptom intensity, realizing that symptoms cannot be overcome, and recognizing that their lives were dominated by the problem facilitated help-seeking among 20 Spanish individuals with AN [55]. Participants in this study also credited a desire for a better life, making behavioural and cognitive changes to recover, and spirituality as facilitators for help-seeking.

**Mixed population** Individuals who reached out to NEDIC identified feeling out of control as a reason for seeking help [65]. Conversely, youth with AN or BN reported that a fear of losing control or gaining weight dissuaded them from reaching out for help [84]. Adolescents and young adults meeting subclinical or clinical ED criteria reported that they did not seek treatment because they did not believe the problem was serious enough, denied that it existed, or felt that they should help themselves, and feared being separated from their family [62]. Men with EDs noted their hesitancy to change, entrenchment in their routines, fear of forced intervention, and not wanting to hurt others’ feelings or ‘burden services’ acted as help-seeking barriers [76]. Alternatively, greater belief in the helpfulness of body image programmes, higher personal ineffectiveness, and lower negative affect facilitated help-seeking behaviour among female undergraduate students in Australia [59]. Lastly, the acknowledgement of needing help and having positive expectations about the efficacy of professional support made participants using ProYouth more likely to seek help [66].

##### Life experiences

**Emerging adults** Among 14 emerging adults in ED treatment, experiencing a life change (e.g., starting university) caused some to avoid help-seeking, hoping that this life change will be the ‘fresh start’ needed to get them out of their disorder [90]. Meanwhile, the life change led others to seek help because their symptoms worsened. Similarly, university-age female athletes in recovery for an ED noted that a change in their environment or an improvement in their mood or self-esteem in another area of their lives motivated them to seek help [55]. Having previous help-seeking experience was related to more positive help-seeking attitudes among university-age students [78]. Consequently, holding reservations about treatment quality due to previous experiences with poor support led emerging adults with EDs to not seek support [87]. All six emerging adults in ED recovery in one study reported that they sought help as it was their ‘last hope’ [91].

**Mixed population** Men with EDs reported being better able to recognize their problem and seek help when their ED was no longer private or when they hit a crisis point (e.g., hospital admission) [76]. Reaching a “breaking point” was also found to facilitate help-seeking among female AN patients [83].

##### Knowledge about ED symptoms and presentation

**Emerging adults** An individual’s understanding of the symptoms and presentation of EDs, which is a component of MHL as operationalized by Jorm and colleagues [18, 19], was also found to influence help-seeking intentions and activity. Only 14.5% of 255 female university students correctly identified that the character in a vignette had BN [71]; the 31 respondents who were identified as probable ED cases in this study were even less likely to identify the ED in the vignette. When comparing a vignette about a person with an ED against themselves, 98 undergraduate females were more likely to attribute their own behavior to a general mental health issue rather than an ED [88]. Misperceptions or uncertainty regarding their symptom severity and EDs in general contributed to the lack of help-seeking activity among young women not in treatment [86], university students with untreated ED symptoms [57], and female athletes [89]. Lastly, a group of eight men who sought treatment reported a lack of knowledge about the prevalence and presentation of EDs in men; thinking they were just “eating healthy” or that only women or gay men suffer from EDs. These misperceptions contributed to their delay in seeking treatment and made them feel isolated in the help-seeking process [10].

It is worth mentioning that university students who received psychoeducation about EDs on campus were more likely to seek help for their friend than themselves if they were experiencing ED symptoms [47]. This effect was more pronounced among those who showed higher levels of eating concerns. Students at a mid-sized American university were also significantly more likely to seek help for a friend with an ED than to seek help for themselves if they were experiencing an ED [93]. Of several variables explored, knowledge about EDs was the only one that significantly predicted whether or not students would seek help for a friend with any psychological concern—not only EDs.

**Mixed population** Once again, help-seeking behaviour among men with EDs was negatively influenced by misinformation surrounding EDs, a lack of male-targeted resources, and not knowing where to seek help [76]. Individuals using ProYouth also noted mental health literacy-related barriers as reasons why they did not intend to seek treatment [66].

#### Close others

Family, friends, and close others were reported to influence the decision to seek help among youth living with an ED in 14 of the studies included in this scoping review.

##### Nature of support

**Children/adolescents** Individuals who received pediatric treatment for AN noted that their parent’s support—particularly their mother’s—was crucial in initiating the help-seeking process and helping them through their ED [70]; siblings were an alternative support for those who did not trust their parents. Similarly, one group of adolescents (n = 15) identified their parents’ “failure to act” on their suspicions of an ED as a barrier to help-seeking [94].

**Emerging adults** Concerns and support from, and help initiating treatment by, family and friends about troublesome behaviours or weight loss acted as a facilitator for treatment-seeking among 26 Asian American college women with body image concerns [9], college students showing ED symptoms [45], and emerging adults who previously received ED treatment [90]. Parents were usually the first to notice and confront adolescents who were currently in or completed ED treatment about their symptoms or behaviour, followed by their friends, teachers, and coaches [94]. Treatment-seeking women meeting subclinical ED criteria were more motivated to seek help if they valued their relationship with the person who noticed and challenged their symptoms [86]. Related to this finding, although 39% of emerging adults with EDs in a cross-sectional American sample (n = 130) sought help for themselves, others reported being referred by a friend (34%), relative (7%), members of their ED program (5%), health center physicians (5%), academic advisors (4%), and residence life staff (4%) [53].

**Mixed population** Encouragement from friends was identified as one of the primary reasons for seeking treatment among individuals who screened positive or as high risk for an ED [60]. Even when young men with EDs made the decision to seek help, they reported that their parent assisted them in executing their plan by taking them to their first appointment [76]. Also, living with others—rather than living alone—was facilitative for female patients with AN in their help-seeking process [83]. Lastly, the source of support was found to differ depending on adolescents’ age of AN onset [79]. In this study, patients with an early onset of AN (age 14 or younger) were more likely to be informed about their first treatment facility by people in their social network (e.g., parent or friend), while health professionals were more likely to inform those with a late onset of AN (19 years and older) of their first treatment facility.

##### Communication style

**Children/adolescents** A group of 14 women who developed EDs in their childhood or adolescence noted that positive feedback about weight loss from close others maintained their ED symptoms and was counterproductive in seeking help [85]; negative feedback was hurtful and also increased denial. Alternatively, caring confrontation or comments that were goal-directed, showed concern, and came from a trusted source were most likely to raise the participant’s self-awareness and facilitate help-seeking. Lastly, a cross-sectional sample of British secondary school students reported that they would not reach out to a teacher for help with their ED out of fear that they would not take it seriously, over-react, or fail to maintain their confidentiality by sharing these details with their parents [67].

**Emerging adults** Positive feedback regarding weight loss in the early stages of the ED and vague or generic comments regarding well-being from friends or family later in the disorder were both found to negatively influence help-seeking behaviour [90]. Negative comments and a lack of concern until severe weight loss or health problems occurred acted as a similar help-seeking barrier for female athletes in recovery for an ED [55] and self-diagnosed individuals with ON [48]. Instead, direct confrontation about behavioural or mood changes and explicit statements of concern from friends and family were found to facilitate the help-seeking process [11, 48, 55]. Specifically, being given an ultimatum, such as being removed from sports or school, brought athletes out of denial and initiated their recovery efforts [55].

In addition to explicit conversations, receiving emotional support and understanding from close others also facilitated emerging adults with ED symptoms to reach out for help [11, 86]. Similarly, female athletes with EDs were less inclined to seek help when they perceived a lack of emotional support from family, coaches, friends, and partners, fearing that these groups did not actually know how to deal with them [89]. Among individuals with BN symptoms, having a support person that had lived experience of mental illness, treated the individual’s symptoms seriously, did not try to fix their problem, and avoided normalizing body dissatisfaction motivated them to seek help [86]. Help with registration, scheduling, or attending appointments from friends or family facilitated help-seeking activity among individuals with EDs [90].

**Mixed population** Regular, open, and calm exchanges (e.g., words of encouragement) and persistence in making ED symptoms the topic of conversations by family and friends were identified facilitators for treatment initiation among individuals in this sample [83].

#### Others with EDs

Exposure to others with EDs served as both a barrier and a facilitator for help-seeking among individuals with EDs.

##### Emerging adults

Although interactions with others with EDs facilitated help-seeking for some university-age female athletes in ED recovery, as it provided a sense of universality, others reported these interactions as a help-seeking barrier—due to the mixed messaging they were now receiving or harmful competitiveness with others with EDs [55]. Notably, one emerging adult with an ED in a qualitative study gained more trust in their GP after their GP disclosed to them that they had lived with BN [91].

##### Mixed population

Similarly, female patients with AN noted that exposure to positive role models who went through treatment as a facilitator due to their potential to motivate, and answer any questions about, treatment initiation [83]. Members of this sample also noted that the presence of individuals with existing EDs in their lives was a barrier for seeking help, as they strongly supported the thin ideal.

#### Healthcare

Twenty-four studies in this scoping review highlighted healthcare-related factors as influences on help-seeking behaviour among youth experiencing ED symptoms.

##### Clinical response to ED symptoms

**Children/adolescents** Although primary care plays an important role for the early detection of pediatric EDs [69], two qualitative studies revealed that GPs were either unable to detect initial ED warning signs [85] or dismissed parental concerns [94]. In one of these studies, young female participants with EDs reported being referred to specialist care only when their physical symptoms were very serious [85]. Of note, individuals from this sample also reported feeling increasingly positive about counselling after successful treatment experiences, while the reverse was true after negative or unsuccessful treatment experiences.

**Emerging adults** Primary care providers have been reported to lack knowledge about EDs, dismiss concerns, and not be able to identify the warning signs of an ED [48, 50, 91]. Other healthcare providers (e.g., psychiatrists, counsellors) were also considered ill-equipped to identify or treat EDs [91]. American university students avoided discussing eating and body image concerns with their GPs because they were not prompted about these issues, there was insufficient time during appointments, they were scared or uncomfortable discussing these topics, or they had closed-off and non-empathetic providers [50]. A lack of trust in providers was also found to dissuade emerging adults from seeking treatment for their ED [50, 58, 90]. An added difficulty cited was that individuals with EDs often present to healthcare providers with other mental or physical health concerns as their presenting issue, whether this was intentional or not [52, 73, 90]; this acted as a barrier for receiving treatment as it delayed ED recognition. Lastly, a group of undergraduate students with weight concerns were more likely to cite that the group format of the intervention was a deterrent for seeking treatment [59].

##### Healthcare-related facilitators and barriers to seeking treatment

**Emerging adults** Outside of the quality of the care provided, emerging adults with EDs reported help-seeking barriers related to the nature of healthcare today, including cost of treatment [54, 58, 87], the lack of access to treatment supports or resources [86, 87], and limited availability of professional treatment [9, 45, 55].

These health sector-related barriers were accentuated for men and ethnic minorities [10, 77]. Men in ED recovery reported that having to negotiate treatment, being misdiagnosed and misunderstood, and the lack of resources and treatments tailored to them negatively influenced their help-seeking behaviour [10]. Additionally, college-age ethnic minority individuals with eating concerns were asked significantly less frequently about their eating behaviors by medical and mental health professionals than non-ethnic minority individuals [77].

**Mixed population** Being knowledgeable about EDs, making confident healthcare recommendations or referrals, and offering regular monitoring were qualities in a doctor identified as help-seeking facilitators among a mixed-age sample of female patients with AN [83]. Young AN and BN outpatients reported feeling more comfortable when seeking help if they could still be involved in treatment decisions [84]. Long wait or referral times and limited availability in the primary care setting were reported barriers for treatment-seeking [83, 84]. The opposite was true during the COVID-19 pandemic—the lack of access to treatment during this time led individuals seeking ED support to reach out to NEDIC [65]. Negative experiences with healthcare providers dissuaded men with EDs from seeking help in the future [76]. Similar to the previous findings, patients presenting to healthcare settings (e.g., emergency department, primary care) with seemingly unrelated concerns, such as abdominal pain or other gastrointestinal-related problems, contributed to a delay in obtaining appropriate help [63, 83].

#### Society

Across 16 papers included in this scoping review, various societal influences played a role in help-seeking behaviour among children, adolescents, and emerging adults.

##### Children/adolescents

Fear of facing discrimination or stigma because of their ED was identified by former pediatric ED patients as a reason for delaying seeking help from a physician [95]. Additionally, negative cultural attitudes towards counselling were mentioned as a barrier for seeking help during adolescence by women who grew up in Asia [85].

##### Emerging adults

Across multiple studies, stigma, shame, and embarrassment surrounding their ED were reported by emerging adults as a barrier to seeking support [45, 46, 54, 58, 86, 87, 90]. Additionally, the media and Western culture’s emphasis on thinness and dieting were identified in two studies as a destructive influence that contradicted emerging adults’ motivation to seek help or to approach recovery [51, 91]. Instead, a group of Australian women not receiving treatment for their ED symptoms reported being able to hide their practices within normative ideals surrounding food, body, or exercise, such as having a food allergy or intolerance, following a special diet, or over-exercising [51]. Lastly, Asian American emerging adults with body image concerns reported the neutral or negative stigma towards mental illness held by Asian culture, and manifested by family members, dissuaded them from seeking treatment or delayed their help-seeking process [9].

##### Mixed population

Mixed-age samples with EDs also reported that their fears and experiences of being labeled or judged and holding a general stigma towards seeking help for their ED negatively influenced their help-seeking process [62, 64, 66, 83]. Comparing oneself to unrealistic body ideals on social media was also mentioned as a barrier for seeking help [83].

## Discussion

To our knowledge, this is the first scoping review exploring healthcare utilization patterns and factors influencing help-seeking behaviour among children, adolescents, and emerging adults with EDs. Our review identified 62 studies that contained a range of research methodologies including 40 cross-sectional studies, 15 qualitative studies, two longitudinal studies, two mixed methods studies, two open trials, and one case report. Of these, 13 articles studied samples of children or adolescents (< 18 years), 37 involved emerging adults (18–25 years), and 12 included a mixed population of both groups.

Many of the included studies reported on rates of intended or actual help sought from parents, friends, and health professionals among individuals with EDs. Although the study samples varied largely—from general samples of university students to pediatric patients in an inpatient ED program—most individuals who met criteria for a clinical or subclinical ED were not receiving help and did not intend to seek help, despite the seriousness of their illnesses. It is critical that help-seeking rates improve so that youth are diagnosed and treated for their disorder sooner, as early intervention for those with ED symptoms has demonstrated a more rapid and lasting recovery compared to delayed intervention [96, 97].

Several studies also reported upon young people’s preferences for who they would like to seek help from, who they actually sought help from, and how helpful each source was for them. Sources of help that were commonly mentioned were close others (e.g., family, friends) and healthcare providers (e.g., mental health professionals). Individuals with EDs identified healthcare professionals—particularly primary care providers—as being unhelpful in diagnosing, treating, and referring them to specialist services. Given that young people with EDs often approach their primary care providers for help or guidance, it is imperative that they develop their ED-MHL by becoming aware of signs and symptoms of EDs, available specialized programs in their area to refer patients to, and how to medically manage patients who are transitioning between ED services. In order to build literacy, confidence, and capacity, it is also recommended that primary care providers receiving training on how to approach and navigate interactions and conversations with youth—and their families—who present to their practice with a potential ED. One American study, comparing teaching modalities (active- vs. print-learning), explored the efficacy of an educational program geared at pediatric care providers regarding the diagnosis and management of EDs [98]. Primary care providers trained in the active-learning group screened significantly more patients for an ED post-intervention, had greater ED knowledge scores, and expressed increased comfort in diagnosing EDs. Employing similar early intervention educational efforts for primary care providers in other settings could lead to earlier diagnosis and treatment of EDs.

A small subset of studies discussed stakeholders who sought help on the behalf of youth affected by EDs [53, 70, 76, 79, 90]. Most notably, parents were identified as bringing their children experiencing ED symptoms to see their primary care provider. The initiation of the help-seeking process by members in one’s close circle is sometimes necessary given that young people with EDs credit their denial about the reality of their symptoms as playing a role in not seeking help [11, 37, 39, 46, 51, 62, 87, 89]. There are many resources available for educating and empowering families to advocate for their children who are at risk or showing early symptoms of an ED; some include Kelty Eating Disorders (www.keltyeatingdisorders.ca), F.E.A.S.T (www.feast-ed.org/), and NEDIC (www.nedic.ca/). However, many caregivers of people with EDs report having unmet needs, including a lack of information about what treatment is available and needing help with coping strategies for themselves [99]. It is encouraged that these resources, and those informed by youth with EDs in the future, be circulated in a targeted manner by healthcare teams or online information centres, to families who suspect their child of having an ED.

Multiple factors related to the individual, including demographic variables, characteristics of the ED, attitudes, beliefs, and fears held by the person affected, and life experiences were found to influence help- or treatment-seeking behaviour. Men and ethnic minorities were less likely to seek help for themselves, due to stereotypes held about the types of people typically affected by EDs (e.g., only teenage girls have EDs). Of note, men and ethnic minorities have been reported to hold less favourable attitudes toward seeking psychological help for many other mental health issues [7, 100], indicating that this finding may not be unique to individuals with EDs. Many of the attitudes, fears, and beliefs held by individuals considering treatment also influenced their decision to avoid treatment, such as wanting to deal with it alone or denying its existence or severity. A low perceived need for help, a preference to manage their problem alone, and a fear for asking for help were some of the attitudinal barriers that were also found to inhibit individuals with common psychiatric disorders, such as depression and anxiety, from seeking treatment [7, 16]. Additionally, misperceptions and uncertainty regarding one’s own ED signs or symptoms were found to contribute to the lack of help-seeking activity among many youths experiencing these symptoms—notably among males [10, 57, 66, 77, 86, 89]. Given that knowledge regarding symptoms and signs of a disorder are considered to be part of MHL [18, 19], these findings indicate that a lack of MHL negatively influences help-seeking behaviour which is a pattern also found among individuals living with common mental disorders [7, 16, 17]. One way to alter these isolating attitudes regarding help-seeking and lack of awareness of developing signs of an ED is by improving ED-related MHL (ED-MHL) from a young age to normalize seeking help for disordered eating prior to an increase in ED severity. In one of the papers included in this scoping review led by Kästner and colleagues, the authors recommend that health education in schools should inform students about EDs and their consequences, while also communicating that there is no need to feel guilt or shame when seeking treatment [83]. The use of mental health curriculums in Canadian schools have been shown to increase knowledge and reduce stigma among high school students [101–103]. These ED-MHL programs can be considered both a health promotion initiative, given that they would be facilitated and received by school children and staff, and also an early intervention effort, given that at-risk youth would also be in attendance and could potentially utilize this information to help themselves. Future efforts directed at improving ED-MHL may improve rates of help-seeking behaviour among those experiencing early signs and symptoms of EDs. However, this must be balanced with the risks of providing such education; given that peer contagion is a risk among youth with EDs [104]. The implementation of such a program should be carefully executed to avoid influencing or triggering ED behaviours among at-risk youth, such as focusing this content on appropriate eating, exercise, social media habits, and media literacy, while increasing self-esteem and body positivity, as demonstrated by ProYouth—an online ED prevention and early intervention program [66].

Factors relating to the relationships between youth with EDs and their family, friends, and others in their close community were also highlighted in the included studies. Firstly, support from one’s family was identified as crucial to an individual’s willingness to seek help for themselves. Conversations where the ED was directly confronted were more likely to influence those with EDs to seek help over generic comments or check-ins about their well-being. Children, adolescents, and emerging adults all benefitted from emotional (e.g., words of encouragement) and formal support (e.g., preparing for or attending appointments) from close others. Early conversations with direct confrontation of the issue and showing support through one’s actions were identified as the most helpful ways that family and friends can motivate individuals with EDs to seek help. Resources informed by these findings should be developed and made available for families and friends of individuals presenting with ED symptoms.

Several factors associated with healthcare services were critical to one’s decision to seek help or continue pursuing treatment for their ED. As stated above, primary care played an important role for many in getting their ED treatment journey started, yet many were reported to lack knowledge about EDs and dismiss the concerns of youth and their parents. The dearth of clinical response to ED symptoms experienced by youth can also be considered a dearth of MHL among primary care providers. A focus on primary care provider education about EDs would allow for earlier identification and would empower healthcare providers to refer their at-risk patients to specialist programs. One example of such an early intervention program is the First Episode Rapid Early Intervention for Eating Disorders (FREED) service model, which has demonstrated real-world validity across diverse clinical contexts in the United Kingdom and Australia [96, 97, 105]. It is important to note that even after youth sought help, they were faced with long waitlists to receive publicly funded specialist treatment or private care was too expensive to afford. It would be ideal to increase the number of ED-specific treatment programs that are accessible, affordable, and available for youth with EDs [29].

Virtual intermediate supports were found to motivate individuals with EDs to pursue treatment. Specifically, moderated online discussion boards, virtual prevention programs, and widely available online screening tools were identified as help-seeking facilitators for youth experiencing ED symptoms. Investment in these in creating and raising awareness about these existing resources is recommended so that individuals who are concerned about their or others’ eating habits can easily access and utilize them, such as by advertising them through mental health literacy curricula at schools.

Finally, cultural and societal ideals were also found to influence help-seeking behaviours among youth with EDs. Stigma, shame, and embarrassment about one’s ED symptoms and seeking help for them were most frequently identified, followed by pressures from social media, diet culture, and culture-specific help-seeking hesitancy. In their paper, Rother and Buckroyd suggested that parents, especially mothers, should receive education about EDs given that they were reported as being instrumental in initiating disordered thoughts by talking about dieting or weight loss with their child from a young age [91]. Kästner and colleagues also recommend greater awareness from other key identified players in the lives of youth with EDs—teachers, doctors, and fitness trainers—in order to break the stigma about reaching out for help [83].

Multiple recommendations to further research and integrate MHL efforts into health promotion and early intervention approaches have been made in this discussion. For this, it is important to acknowledge that the reason for a lack of widespread health promotion initiatives and research is because there is little consensus—particularly between sufferers, the public, and health experts—on what its content should entail [20, 106], To address this subjectivity, it is recommended all differing perspectives on the issues are presented in these health promotion or education programs to fully inform the reader [20, 22, 107]. Although it is not easy to mitigate the effects of these societal barriers, a shift in thinking is possible if health promotion and early intervention efforts are focused on altering thoughts and behaviours for help-seeking for EDs at individual, familial, and healthcare levels.

### Strengths

We used a rigorous and evidence-based methodology for our scoping review, which included a thorough review of literature from seven databases, without language restrictions, and few exclusion criteria. Several papers were translated into English for full-text review and the references of included reviews and book chapters were reviewed to ensure other relevant studies were not missed. Additionally, the mixture of qualitative and quantitative methods in the selected studies offered generalizability and in-depth understanding of the nature of help-seeking among young people with EDs.

### Limitations

Although the onset of EDs typically occurs in adolescence, very few papers specifically explored help-seeking behaviours among children and adolescents. Some of the included studies collected data from parents or healthcare professionals involved in the youth’s care, but it was difficult to separate the results offered by the individuals with EDs from these other individuals, and we were unable to draw conclusions from these differing groups.

While our goal was to explore help-seeking behaviour among children/adolescents (< 18 years) and emerging adults (18 to 25 years), the age ranges of samples of the included studies did not always align with our set age criteria. We decided to create the mixed population category to accommodate studies whose age range overlapped the boundary between children/adolescents and emerging adults. Additionally, many of the studies included individuals over the age of 25. However, the authors of this review considered it necessary to include these studies given that all relevant findings could inform our understanding of this phenomenon. However, the results and subsequent conclusions drawn from each of the age groups may overlap.

The findings yielded from this exploration identified other important initiators and supporters of individuals seeking help for their ED, including family, friends, and healthcare providers. For this reason, it is possible that other aspects of help-seeking behaviour among youth with EDs were not addressed in this review, as it only explored the perspectives of youth with lived experience of an ED on their help-seeking attitudes and activity. It would therefore be useful to explore the perspectives of other people involved in the treatment-seeking process to further inform factors that influence decisions to seek help.

## Conclusions

This scoping review presents findings related to the help-seeking activities and attitudes of children, adolescents, and emerging adults with EDs. Youth who have sought treatment for their ED credit multiple motivational influences for reaching out for help, such as increased severity of ED symptoms, explicit support from close others, and helpful experiences with medical and mental health providers. However, the findings regarding help-seeking rates in this review make it clear that many young people experiencing ED symptoms are not reaching out for help, with some of the documented reasons for not doing so including inaccurate preconceptions about EDs, a lack of affordable and available ED treatment options, stigma about EDs and help-seeking, as well as body ideals enforced by social media.

Many of the insights offered in these studies can be used to inform future educational resources and healthcare policies aimed at individuals with EDs, members of their close communities, and their healthcare providers. Future studies that explore help-seeking behaviour in youth with EDs should focus on gaining the insight of populations who are not coming forward for support, such as individuals with an ED other than AN or BN as well as underrepresented populations with EDs (e.g., men, ethnic minorities); this suggestion has also been put forward by other researchers in the field [20]. Additionally, researchers are also encouraged to use mixed methodology when studying this topic, given that mixed methods allow researchers to incorporate the strengths of both quantitative methods (generalizability, lack of bias), and qualitative methods (in-depth understanding of contextually-influenced phenomena) [108]. Although this study focused on the experiences of youth with EDs, it would also be beneficial to retrospectively explore the help-seeking experiences of adults with lived experience of an ED as well as caregivers. Lastly, there is a need for more research efforts dedicated to assessing health promotion and early intervention programs designed to educate various stakeholders or members of the public on symptom recognition or treatment benefits, as this topic has received little to no attention in the ED, and general psychiatry, literature despite the potential they hold to increase help-seeking behaviour [7, 16, 20]. For instance, the MHL paradigm can be used as a framework for this research as it has the potential to inform health promotion and early intervention efforts for family members, friends, and others who interact with youth with EDs [106].

## Supplementary Information

**Additional file 1.** Extracted data from all 62 articles included in the scoping review, organized by age group.

## Data Availability

All data is published and in the public domain. Data sharing is not applicable to this article as no datasets were generated or analysed during the current study.

## Abbreviations

AN: Anorexia Nervosa
BED: Binge Eating Disorder
BN: Bulimia Nervosa
EAT-26: Eating Attitudes Test
ED: Eating disorder
ED-MHL: Eating disorder-related mental health literacy
EDE-Q: Eating Disorder Examination-Questionnaire
FREED: First Episode Rapid Early Intervention for Eating Disorders
GP: General practitioner
NEDA: National Eating Disorders Association
NEDIC: National Eating Disorder Information Centre
NEDSP: National Eating Disorders Screening Program
MHL: Mental health literacy
ON: Orthorexia Nervosa
