# Supplementary material for: Help-seeking attitudes and behaviours among youth with eating disorders: a scoping review
Source: J Eat Disord. 2022 Feb 14;10:21. doi: 10.1186/s40337-022-00543-8 (PMC8845232; doi:10.1186/s40337-022-00543-8)
Supplement: Supplementary file 1 — Additional file 1. Extracted data from all 62 articles included in the scoping review, organized by age group. [file 40337_2022_543_MOESM1_ESM.docx]

**SUPPLEMENTARY FILE 1:** Extracted data from all 62 articles included in the scoping review, organized by age group. **Table 1.** Eligible studies with samples of children and adolescents (age range included participants ages 18 or younger) (n=13)

| **Reference** | **Study Type** | **Sample Size** | **Sample Description** | **Mean Age (SD) [Range]** | **Help-Seeking related Outcomes Reported** | **Type of Analysis** | **Help-Seeking related Findings** |
| --- | --- | --- | --- | --- | --- | --- | --- |
| Bühren et al., 2017 | Cross-sectional study | n=258 | Children and adolescents with (Atypical) Anorexia Nervosa receiving inpatient treatment | 14.7 (1.8)  [up to age of 18] | Type of medical or psychological speciality the patient first presented because of the ED; time elapsed between ED onset and first presentation; number of physicians seen from different disciplines; number of outpatient consultations; length of time patients underwent outpatient treatment prior to hospitalization | Descriptive statistics (continuous variables); exact numbers and proportions (nominal variables) | Most patients had first seen a pediatrician, child/adolescent psychiatrist, or a general practitioner. Primary care played an important role for early detection of AN. On average, 6 months elapsed between first ED symptoms and first presentation in primary care. From there, another 7 months (on average) elapsed between first presentation at the GP and hospitalization for AN. |
| Ciao et al., 2020 | Qualitative study | n=15 | Adolescents with an ED (and their caregivers) attending (or recently completed treatment at) one of three ED treatment centres in the United States of America | 15.20 (1.81) [12-18] | EDE-Q; interview questions addressed experiences identifying and responding to the ED (e.g., “Did anyone around you know about the eating disorder? What did they do or say?”, “Based on your own experience, what advice do you have for family members who want to encourage their loved ones to get help?”) | Thematic analysis and coding of interview transcripts | 60% identified parents (typically mothers) as the first to notice their ED; others included friends of adolescent, teachers, and coaches. 87% of adolescents stated that parents were typically the ones to confront them about changing their behaviour. 53% of adolescents report weight loss and thinness as their earliest symptoms. As a barrier, 60% of adolescents stated that other’s (usually parents’) hesitancy to act on suspicions was a barrier to help-seeking. 42% of the time, parents’ concerns were dismissed by their child’s pediatrician or health care provider. |
| Fatt et al., 2021 | Cross-Sectional Study | n=1002 | Australian school students who met DSM-5 criteria for an ED (from first wave of the Every BODY study) | 15.14 (1.4) | EDE-Q; self-identification with a body image problem; help-seeking for a body image problem; Kessler Psychological Distress Scale (K10); social functioning subscale (SF15) of Paediatric Quality of Life Scale (PedsQL) | Weighted chi-squared analyses; binary logistic regression analysis (relationships between self-identification and predictor variables, such as help-seeking | Participants were more likely to self-identify as having a body image problem if they were female. Self-identifying adolescents were 2.71 times more likely to seek help for a body image problem. Around 10% of the sample sought help/treatment. |
| Fatt et al., 2020 | Cross-sectional study | n=1002 | Australian school students who met DSM-5 criteria for an ED (from first wave of the Every BODY study) | 15.14 (1.4) | ED diagnosis, EDE-Q (Weight and Shape subscale); help-seeking behaviours “for a body image problem”; Kessler Psychological Distress Scale (K10); Pediatric Quality of Life Scale (PedsQL) | Descriptive statistics; univariate analyses comparing those who had sought help and chi-square tests for categorical variables; multivariate binary logistic regression to assess sex, BMI, ED diagnosis, and help-seeking | 10.1% of participants reported having sought help for a body image problem. Most adolescents who did seek help accessed GPs/doctors, with less receiving specialist care from psychiatrists or therapists. Increased likelihood of help-seeking was associated with female sex, sexual minority status (non-heterosexual), being born outside Australia, older age, having a major ED (compared to having an unspecified or other specified feeding or other ED diagnosis), higher self-reported psychological distress, and self-reported reduced psychological and social functioning. |
| Forrest et al., 2017 | Cross-sectional study | n=281 | A nationally representative sample of U.S. adolescents from the National Comorbidity Survey Replication Adolescent Supplement who were recruited between 2001 and 2004 and have an ED | [13-18]  Ages 13-14:  82  Ages 15-16: 117  Ages 17-18:  82 | Characteristics of EDs (e.g., type, behaviours, impairment); age at which they last experienced ED symptoms; Sheehan Disability Scale; psychiatric comorbidities; help-seeking or treatment history for emotional or behavioural problems | Calculated proportion of seeking ED treatment among adolescents with any ED overall and within subgroups (e.g., by ED characteristics). When comparing treatment seeking across subgroups, estimates of the treatment-seeking ratio with 95% confidence intervals were reported. | 20% of adolescents sought ED treatment. In terms of seeking treatment: females were 2.2 times more likely than males; older adolescents (17-18 years) were 4.4 times more likely than younger adolescents (13-14 years); adolescents with at least one parent with a college degree were 1.8 times more likely compared to those without; adolescents with AN or BN were 2.4 and 1.9 times more likely than those with BED; those who admitted they restricted and purged were 1.9 and 2.9 times more likely; those who admitted moderate or severe ED impairment in the last 12 months were 5.5 and 10.4 times more likely compared to those denying ED impairment; those who utilized any services for treatment of an emotional or behavioral problem were 1.7 times more likely compared with adolescents who had not utilized any services. |
| Kendal et al., 2017 | Qualitative study | n=400 messages posted to online forum | Users of a moderated online discussion forum run by a pro-recovery ED charity to receive support from others around disordered eating | Not reported. Based on quotes included in paper, individuals younger than 18 were involved. | Ways in which young people are using the online discussion forum for support of EDs | Thematic analysis of discussion forum posts | Forum users offered mentorship in the form of advice and encouragement (e.g., drawing from their own lived experience). The forum seemed to be a trusted, friendly environment for individuals to discuss fears in a place of mutual support. Anonymity offered support that would be difficult to find in face-to-face encounters. Forum was flexible in responding to individual needs at different points in the journey (e.g., always open, multiple users can help). The forum can be used to receive help (e.g., a healthcare provider suggested that they try the forum) or to find help (e.g., members of the forum recommend different treatments). |
| Knightsmith et al., 2014 | Cross-sectional study | n=511; 38% (n=195) currently or previously had an ED | Adolescents in UK secondary schools | 15.4 (2.3)  [11-19] | Student experiences of ED in school; perceptions of how their school could support students with ED; ideas about how schools could become more supportive | Content analysis was used to analyze students’ responses | Only 7% (n=33) of students would talk to a teacher if they were concerned a friend might have an ED. Many students fear that teachers would not take it seriously, over-react, or fail to maintain confidentiality (e.g., would inform parents). If they were to tell a teacher, 73% (n=337) would prefer to discuss ED concerns face to face as opposed to in writing/over e-mail (22%, n=102) or text (5%, n=22). Students perceived being able to receive support from non-teaching school staff (e.g., counsellors) as helpful. |
| Maier et al., 2014 | Cross-sectional study -Retrospective* | n=36 | Former in- and outpatients of a major pediatric mental health service who had previously been diagnosed with AN and who completed the survey | Age at survey: 19.3 (2.0)  [15-24] | Questionnaire on stigmatization in patients with anorexia nervosa (QSAN: patients’ experiences with stigmatization and discrimination and course of illness) | Descriptive analysis | Participants felt they received the most criticism and the most support from parents. 31% reported they waited a considerable amount of time before visiting a physician due to fear of being criticized and blamed. 34% of participants waited to undergo treatment due to fear of being excluded or degraded. Average period between AN onset and first contact with a physician was 8.2 months. Average delay between the onset of AN and the start of treatment was 9.3 months. |
| McNicholas et al., 2018 | Cross-sectional study | n=290 | Youth from secondary schools in Ireland | 16.8 [15-19] | Participants’ attitudes and experience regarding EDs; level of body satisfaction; knowledge of how to access ED information and health services; preferred help-seeking behaviours | Principal components analysis with Varimax rotation; two-way mixed ANOVA with Bonferroni corrections (e.g., the influence of type of help and personal eating concern on the likelihood of seeking help) | 99 were concerned about their own eating habits and of these, 51 discussed these concerns with someone: family (n=30) or friend (n=17). Very few reached out to a health professional or a support organization (n=4). Lack of disclosure due to fear of being judged, felt problems were not sufficiently serious, not knowing who to tell (i.e., poor understanding of help-seeking options), low levels of confidence in how to access professional help. All reported that they would seek support from personal contacts first, then information providers/media, and finally from mental health experts, if they developed eating concerns. Those who reported eating concerns were significantly less likely to seek help compared to those without concerns. |
| Meyer, 2001 | Cross-sectional study | n=238 | High school junior and senior females from the USA | 17.8 [15-19] | Questionnaire for ED Diagnoses (Q-EDD; symptoms of EDs); Attitudes Towards Seeking Professional Psychological Help-Short Form (ATSPPH-SF; measures attitudes towards traditional counselling services) | Differences in treatment-seeking between groups was examined using chi-squared tests and ANOVA. | 16% (n=38) met ED clinical criteria; 33% (n=78) symptomatic (met subclinical criteria for EDs). 98% were not in therapy, primarily due to belief that their behaviours were not problematic enough to merit counseling, not believing they have a problem, and not wanting anyone to know. Participants who met clinical ED criteria reported that a fear of disclosure to others kept them from treatment. Symptomatic participants were significantly more likely (compared to the ED group) to deny that their eating concerns merited counselling; however, ED participants were significantly more reluctant than symptomatic participants to let others know about their problem. |
| Schoen et al., 2012 | Qualitative study – Retrospective* | n=14 | Female university students with a current or previous diagnosable ED | Age at interview:  23.0 [18-30]  Age at diagnosis: 14.1 | Participants’ self-reported process in seeking psychological help for their ED | Grounded theory; open and selective coding methods | Participants often weaved in and out of awareness and denial about the severity of their ED, the meaning of an ED, and their need for support. A defining or ‘snapping’ moment or critical health incident jolted them out of denial. Positive feedback about weight-loss maintained ED and was counterproductive in seeking help, while negative feedback was hurtful and increased denial. Caring confrontation, or comments that were goal-directed, showed concern, and came from a trusted source, were most likely to raise the participant’s self-awareness. Negative cultural attitudes toward seeking counseling were mentioned by women who grew up in the Middle East and Asia. Participants’ high need for control negatively affected attitudes towards seeking help. Severity of disorder was not mentioned as a contributing factor toward attitudes of help seeking. After successful treatment, participants’ attitudes about counselling became more positive; after unsuccessful treatment, attitudes became more negative. |
| Sparti et al., 2019 | Cross sectional study | n=2298 | Young people within randomly selected households through the Young Minds Matter Survey in Australia who screened positive for disordered eating | 15.45 [13-17] | Disordered eating (DE) and lifetime ED; Diagnostic Interview Schedule for Children Version Four (DISC-IV); Strengths and Difficulties Questionnaire (SDQ; problems with functioning); help received in the past year | Multinomial logical regression (e.g., the association between DE severity and highest level of help received) | 31.6% (n=922) of participants had disordered eating (DE) in the past 12 months. More than 85% of youth with DE reported help-seeking in the past year, commonly self-help; around 40% received help from formal services (school- based, primary care or specialist services). The use of more specialized and intensive services was associated with more severe DE, greater problems with functioning, female gender, and 12-month mental disorder or subthreshold mental disorder symptoms. |
| Tierney, 2008 | Qualitative study – Retrospective* | n=10 | Individuals treated for AN as teenagers (11-18 years of age) who received both inpatient and outpatent treatment | 17 (1.8) | Participants’ treatment experiences | Thematic analysis with coding | Participants were often made to see their GP by their parents when parents noticed behaviour or physical changes. It was common for GPs to fail to detect initial ED signs. Even if diagnosed early, a lack of appropriate specialized services acted as a major obstacle. Participants were often first seen by community practitioners lacking experience in EDs. They were often referred to specialist care only when physical symptoms progressed to a very serious stage. Parents, particularly mothers, were noted as crucial in helping participants through their ED. Siblings were an alternative support for those who did not trust parents. |
| *Participants are retrospectively reflecting on their experiences when they first sought treatment. Their age at the time of the study does not reflect the age related to their responses about help-seeking. | | | | | | | |

**Table 2.** Eligible studies with samples of emerging adults (age range included participants ages 18 to 25 or older) (n=37)

| **Reference** | **Study Type** | **Sample Size** | **Sample Description** | **Mean Age (SD) [Range]** | **Help-Seeking related Outcomes Reported** | **Type of Analysis** | **Help-Seeking related Findings** |
| --- | --- | --- | --- | --- | --- | --- | --- |
| Akey et al., 2013 | Qualitative study | n=34 | American individuals with EDs who were recruited as part of a larger study on the management of EDs | 25 (8.3)  [18-53] | Eating Attitudes Test (EAT-26); experiences managing their disorder; reasons why they did not seek help | Grounded theory approach and data were analyzed using latent content analysis and constant comparative techniques | Participants reported not seeking social support for their ED for the following reasons: 1) it was not perceived as needed (denial); 2) they believed that their disorder was low severity or that they were fine; 3) they held reservations about the quality of the support due to experiences with poorly conceived or developed support; 4) various perceived barriers (i.e., lack of access to supports, cost, self-preservation, stigma, and concern for others (family) to shield them from the pain that ED treatment would cause; and 5) participants were unable to articulate their needs (low self-efficacy) and withdrew socially. |
| Ali et al., 2020 | Cross-sectional Study | n=291 | Australian young adults who responded to an ad that said: “Do you worry about your eating, weight, or body shape?” | 20.04 (2.02) [18-25] | Weight Concerns Scale (WCS); Short Evaluation of Eating Disorders (SEED); EDE-Q; Attitudes Toward Seeking Professional Psychological Help-Short Form (ATSPPH-SF); General Help-Seeking Questionnaire (GHSQ); Actual Help-Seeking Questionnaire (AHSQ); Barriers Towards Seeking Help for EDs Questionnaire (BATSH-ED) | Differences in demographics and past help-seeking behavior between ED symptomatology groups explored using Fisher's exact test for categorical variables and one-way analysis of variances for continuous variables. | Despite the belief that help-seeking is useful, only a minority of participants with elevated symptoms (e.g., AN, BN, BED), believed they needed help. Most frequently cited barriers to seeking help for ED symptoms: concern for others (don't want others to worry about my problems), self-sufficiency (I should solve my own problems), fear of losing control over their weight/eating, denial, failure to perceive the severity of the illness, stigma, and shame. 60% of the entire sample reported that they would seek help (e.g., mental health professional, family). More than one-third of the sample reported they had sought help for eating, weight, shape concerns in past (20% sought help from a mental health professional and 13-15% from GP, parents, websites, friends, partner). |
| Arthur-Cameselle & Quatromoni, 2014 | Qualitative study | n=16 | Female athletes from an American University who experienced recovery from an ED who participated in a larger study | 20.7 (2.4)  [18-25] | Factors that initiated a desire to change disordered eating behavior; factors that assisted recovery efforts; factors that hindered recovery efforts | Content analysis, inductive coding, realist approach | Factors initiating motivation to change eating behaviours and attempt recovery: experiencing negative (physical) consequences of the ED, inability to compete in sport, confrontation or intervention brought them out of denial, desire for a better life, improvement in mood or self-esteem in another area of their lives, opening up to others, and a change in environment. Factors that assisted in recovery efforts (e.g., help-seeking): making cognitive changes to recover, supportive relationships, receiving professional help, making behavioural changes (avoiding triggers), aspects of sport environment, interactions with others with EDs (sense of universality), medications, and spirituality. Barriers to recovery: lack of support from important others (direct negative comments, lack of concern), difficulty finding professional care, interactions with others who had active EDs, negative thoughts, relationship conflicts (break-ups), negative emotions and low self-esteem, pressures to excel in sport/society. |
| Atkinson & Wade, 2013 | Cross-sectional study | n=121 | Female first year undergraduate students from a South Australian university | 19.3 (1.55) [17-25] | Eating Disorders Inventory (EDI; Body dissatisfaction and ineffectiveness subscales); EDE-Q (Weight and shape concerns subscales); Positive and Negative Affect Schedule (PANAS; negative affect); Difficulties in Emotion Regulation Scale (DERS); Ways of Coping Questionnaire (WOC; escape-avoidant subscale) (WOC); Weight Concerns Scale (WCS); participants randomized to the motivational enhancement exercise were asked questions about the thin-ideal | Frequencies (reasons for participation); linear multiple regression analysis (each dependent variables interest and likelihood to ascertain predictors of participation); 1-way MANCOVA (differences for motivational enhancement compared to controls on participation) | Interest in participating in and likelihood of contacting the researcher to partake in ED prevention (body image interventions) were low overall; lack of time was the most endorsed reason. Participants high on weight concerns were more likely to cite the group format of the intervention as a deterrent. A greater belief in the helpfulness of body image programmes, higher personal ineffectiveness, and lower negative affect were significant predictors of interest in participation. There was no significant difference between those who did and did not undergo the motivational enhancement with respect to interest and likelihood of participation. |
| Becker et al., 2004 | Cross-sectional study | n=289 | Randomly- selected college-age students from the National Eating Disorders Screening Program (NEDSP) who agreed to be contacted in the 2 years after the program and were successfully reached by telephone | 24.7 (10.2) | Participants’ reasons for attendance in the program; responses to the NEDSP; treatment-seeking information | Thematic analysis | Of the participants who received a recommendation for further clinical evaluation n=109), 51% reported they were willing to follow up with this recommendation; 42% were neutral or had little interest and 8% were unwilling. Participants’ reasons for not pursuing follow-up suggestion included: feeling like they could handle the problem on their own (36%), inconvenience (19%), feeling the problem was not serious (15%), expense (13%), feeling there was no problem (12%), already having a therapist (8%), not wanting others to know (8%), unavailability of professional treatment (2%), and no parental support (2%). 35% of participants who were recommended for further clinical evaluation said the screening program was very or extremely important in deciding to seek treatment. |
| Becker et al., 2003 | Longitudinal study | n=289 (Study II only) | Randomly- selected college-age students from the National Eating Disorders Screening Program (NEDSP) who agreed to be contacted in the 2 years after the program and were reached by telephone | 23.57 (10.1) | Self-reported ethnicity; clinician referral and treatment-seeking patterns between ethnic minority and White participants | Descriptive statistics; ANOVA and Scheffe post-hoc comparison tests (e.g., effects of ethnic status on variables of interest); logistic regression (e.g., referral patterns based on ethnicity) | Among participants who acknowledged concerns about eating (N=224), ethnic minorities (9.5%) were less likely to seek ED-related treatment than non-ethnic minorities (27.1%) after participating in the NEDSP. Ethnic minorities stated that they were asked by their doctors about eating behaviors significantly less frequently than were non-ethnic minorities; not to the level of significance, but ethnic minorities were also asked by mental health professionals less frequently about their eating behaviours. Ethnic minorities who acknowledged eating concerns were much less likely to receive a recommendation to see a health professional during the NEDSP screening (31%) than non-ethnic minorities (60%), even though ED symptomology did not differ between groups. |
| Chen et al., 2010 | Cross-sectional study | n=255 | Young female students at a Singaporean university reading a vignette about a fictional female character (“Kelly”) | 19 (1.8) | Participants’ perception of what Kelly’s (character in vignette with BN) main problem is; participants’ perception of which (among multiple) interventions (people, treatments/activities and medicines/pills) would be most helpful in treating Kelly’s problem; participants’ personal experience of an eating problem; EDE-Q | Percentages; Mann-Whitney U-tests (recognition of problem); means of chi-squared tests (e.g., associations between ED psychopathology/ problem recognition and responses to the most helpful interventions, the person most likely to be approached in the first instance, and Kelly’s likely prognosis) | 14.5% of participants correctly labelled Kelly’s problem as BN. Participants identified the following interventions as most helpful to treat Kelly’s problem: consulting a primary care practitioner, counsellor, or psychologist; seeking the advice of a (female) family member or friend; getting advice about diet and nutrition; and taking vitamins and minerals. Participants were less positive about the benefits of a psychiatrist and were ambivalent about the use of psychotropic medication. Participants’ mothers were most often considered helpful as they are an initial source of support. Respondents who were identified as probable ED cases (n=31, 12.2%) were less likely to identify the main problem as an ED. A minority of participants (<30%) believed that treatment would result in full recovery. |
| Coffino et al., 2019 | Cross-sectional study - Retrospective* | Total: n = 622  AN: n=275; BN: n=91; BED: n=256 | American respondents from the National Epidemiologic Survey on Alcohol and Related Conditions III (2012-2013) meeting DSM-5 criteria for lifetime AN, BN, and BED, who answered help-seeking-related questions | [18+] | Diagnostic assessment of EDs; help-seeking behaviors for EDs (e.g., whether they sought help, type of help sought) | Weighted means; cross-tabulations; chi-square tests; multiple logistic regression; ANCOVA | Prevalence of ever seeking help for AN, BN, and BED: 34.5%, 62.6%, and 49.0% respectively. Most frequently used form of help was talking to a counselor, therapist, doctor, or psychologist, followed by use of self-help or support group (similar among men and women). Men and ethnic minorities (non-Hispanic Blacks [NHBs] and Hispanics) were significantly less likely to ever seek help for BED than women or non- Hispanic Whites [NHWs]. Hispanics significantly less likely to seek help for AN and BED, vs. NHWs. Relative to NHWs and NHBs, Hispanics reported significantly later age of first seeking help for AN; NHB and Hispanics significantly later age of first seeking help for BN vs. NHW. Men reported significantly higher prevalence of being hospitalized and prescribed with drugs. |
| Del Valle et al., 2017 | Cross-sectional study | n=20 | Individuals with AN recruited from one of three outpatient clinics in Spain | 24.46 (6.92) | Eating Disorder Inventory-II (EDI-II) (disorder severity); Goldberg General Health Questionnaire-28 (current health status/health-related quality of life); Interview on Help-Seeking for Mental Health (IHS-MH) (e.g., search for professional help as a process with the stages of problem recognition, problem disclosure, and decision to seek professional help) | Descriptive statistics; ANOVAs or non-parametric tests (Chi-squared) were used to explore differences between study groups with Bonferroni post-hoc tests; within-subjects differences were examined using Repeated measures MANOVAs; effect sizes were calculated. | From symptom-onset, participants took a mean of 49.6 months to consult with someone about ED.  37.5% of participants first disclosed symptoms to their closest relatives/partners and 37.5% to their closest friend; first healthcare provider disclosed to was GP. Reasons for delaying treatment: belief that they could control symptoms or would go away, minimizing symptom importance, pleasantness of symptoms, and problem was not dangerous. Most important motivators for help-seeking: symptoms’ interference with daily life, realizing symptoms cannot be overcome, confirmation of the problem’s persistence despite efforts to control it, feared an increase in symptom intensity, ED dominating lives, and needing aid and understanding from those they disclosed to. Participants were more likely to disclose their symptoms to those who were concerned after being directly confronted about their behavioural or mood changes. |
| Dotson et al., 2011 | Cross-sectional study | n=283 | A general sample of undergraduate students from Georgia, USA were recruited through their psychology courses | 19.67 (1.44) [18-25] | Mizes Anorectic Cognitions Questionnaire-Revised (MAC-R) (assessed Disordered eating cognitions); Attitudes toward Seeking Professional Psychological Help | Hierarchical regression analysis was used to explore the relationships between independent variables and various help-seeking variables. | Greater levels of disordered eating cognitions (e.g., fear of gaining weight, perceiving self-worth as related to self-control over diet and weight) were associated with less favourable help-seeking attitudes. Being a female, being a European American, and having previous help-seeking experience were all related to more positive help-seeking attitudes. |
| Eisenberg et al., 2011 | Cross-sectional study | n=2822 | Undergraduate and graduate students enrolled at a large, midwestern, public university in Fall 2005 | [18-30] | SCOFF (ED symptoms); Patient Health Questionnaire-9 (PHQ-9; depression and anxiety); perceived need for and utilization of mental health services; history of receiving counselling or therapy for mental health by a professional | 2-tailed chi-square tests (subgroup differences); descriptive statistics (e.g., co-occurrence of ED symptoms and other health measures) | Of those with positive ED screens (~n=205), 20.4% had received past-year mental health treatment (e.g., counselling or medication) and 48% perceived a need for help. Among those with positive ED screens who did not receive services, their reasons for this were “I have not had any need”, “stress is normal in college”, “the problem will get better by itself”, and “I don’t have time”. |
| Fitzsimmons-Craft, Eichen, et al., 2020 | Open trial | n=61 | American university students who screened positive for AN, provided a referral for treatment, and completed the 9-month follow-up for the Healthy Body Image program | 20.95 (3.34) [18-34] | Participant’s reaction upon receiving the referral for treatment from the Healthy Body Image program; treatment-seeking history/ behaviour; reasons for seeking treatment and from whom; participant-reported ways to increase referral acceptance; barriers to seeking treatment | Descriptive analysis of questions; ANOVAs and chi-square tests to compare changes in ED pathology and psychiatric comorbidity between those in treatment, initiated treatment, or had not initiated treatment | 33% reported already being in treatment at the time they received the referral, 26% initiated treatment since that time, and 41% did not initiate treatment. The most common reasons for seeking treatment were emotional distress, concern with eating, and health concerns. Among those who did not receive any treatment, the strongest treatment barriers reported were believing one should be able to help themselves, believing the problem was not serious enough to warrant treatment, not having time, treatment is too expensive, feeling shame or embarrassment, and a lack of trust in providers. |
| Hackler et al., 2010 | Cross-sectional study | n=145 | Undergraduate students from an American university who met the criteria for disordered eating were recruited through their psychology courses | Not reported [18-25] | EAT-26 (eating attitudes); Self-Stigma of Seeking Help Scale (self-stigma); Attitudes Toward Seeking Professional Psychological Help–Short Form (ATSPPH-SF) (attitudes towards seeking counselling); Disclosure Expectations Scale (DES) (anticipated risks/benefits) | Hierarchical regression analysis was used to explore the unique and combined effects that independent variables had on attitudes towards seeking counselling. | Self-stigma (individual’s view of themselves if they were to seek help for their ED) and the anticipated benefits (of talking to a counselor about a psychological problem) were significantly associated with attitudes toward counseling for people with disordered eating among those who met ED criteria; gender played a moderating role. Self-stigma increased = positive attitudes towards seeking counselling decreased for women; this effect even more pronounced for men. As anticipated benefits of seeking counseling increased, attitudes toward seeking counseling became more positive for women, but were less so for men. |
| Javier & Belgrave, 2019 | Qualitative study | n=26 | Asian American emerging adults at a diverse college who self-reported as having some body image problems | 19.25 (0.78) | Participants’ perspectives of body image; definition of disordered eating behaviours; how body image/dissatisfaction develops among ethnic minority women; consequences of body dissatisfaction and disordered eating among ethnic minority women; barriers to treatment-seeking for EDs among ethnic minority women; what may prevent ethnic minority women from staying in ED treatment programs | Social Constructivist Grounded theory, using the constant comparative method | Having available resources (e.g. nutritionist, finances for treatment, time) and familial support were facilitators for treatment-seeking. Stigmatization of mental illness (e.g. lack of acknowledgment that mental illness exists, viewing mental illness in a negative way) acted as a barrier. Family members of older Asian generations might acknowledge that something is wrong with their daughter, but do not attribute the problem to mental health. Some participants report that family members were the primary individuals who did not believe in mental illness or that mental illness could be treated via methods other than psychotherapy or via psychological counselling. Overall, family was the strongest driving factor for one’s decisions to seek out or remain in treatment. |
| Kaitz et al., 2020 | Cross-sectional study | n=102 | Students from a small private American university | 21 (2.6) [18-35] | EAT-26; communication with healthcare providers; experiences discussing eating and body image issues with their GPs; reasons why they may not have shared concerns; recommendations for what would be helpful for future conversations with their GP regarding this topic | Consensual Qualitative Research (CQR) method was used in the development of a coding rubric and the coding of themes within the narrative data. | Only 35% of this sample communicated their eating and body image concerns with GP. They felt GPs were not informed or understanding of EDs. Not prompted about this topic by their GP was a significant barrier to bringing up eating and body image issues. Communication barriers with GPs: not enough time in appointment, felt issues were not significant enough, preferring specialized mental health providers instead for disordered eating, embarrassment, fear in discussing concerns, and lack of trust. Might share concerns with others (friends/family) instead of GP. |
| Kindermann et al., 2016 | Case report | n=1 | Female experiencing ED symptoms who participated in ProYouth (a virtual ED prevention program) over three months | 18 | Short Evaluation of Eating Disorders (SEED); Weight Concerns Scale (WCS), PHQ-4, satisfaction with ProYouth, actual and intended help-seeking activities during the past 3 months, reasons for not seeking professional help | N/A | After participating for 3 months, the participant reported that ProYouth—specifically the chat session feature—contributed to her decision to seek face-to-face support and start psychotherapy. Also, the participant reported that ProYouth helped her deal with her topic ED for the first time (gaining new knowledge about her eating behaviour and related issues). |
| Lipson et al., 2013 | Cross-sectional study | n=2180 | Random sample of American undergraduate and graduate students | [18+] | Phase 1: Weight Concerns Scale (WCS); EDE-Q (ED symptoms); Treatment barriers were explored among those who had reported no past-year treatment and had a positive ED screen; traditionally emphasized treatment barriers | Phase 1: Basic analyses to examine prevalence of ED symptoms and ED treatment rates; barriers to treatment reported by students with untreated ED symptoms (significance reported using two-tailed chi-square tests). | Phase 1: Of the 28.65% of students with significant ED symptoms or elevated weight concerns (n=657), 86.5% had not received treatment. Students with untreated symptoms who did not seek help (n=558) said this was due to lack of time, lack of perceived need, desire to deal with the issue on own, uncertainty of how serious their needs are, and belief that they do not need treatment. Phase 2: All students were referred to universal online prevention or selective/indicated intervention programs based on ED symptoms; enrolment highest for who were assigned a tailored intervention (18.1%) and lowest for students in the universal prevention (4.1%). |
| Masuda et al., 2017 | Cross-sectional study | n=257 | Asian American college women | 19.68 (2.53) | Attitudes toward Seeking Professional Psychological Help scale (ATSPPH; Stigma Tolerance and Interpersonal Openness subscales); General Health Questionnaire-12 (GHQ-12; psychological distress); Mizes Anorectic Cognitions Questionnaire- Revised (MAC-R; disordered eating cognitions); Acceptance and Action Questionnaire (AAQ-II; psychological inflexibility) | Descriptive statistics; Welch *t-*test; multiple mediation analysis using PROCESS script | Psychological distress, psychological inflexibility, and disordered eating cognitions were all significantly positively associated with one another and were all significantly negatively associated with help-seeking stigma tolerance (one’s ability to tolerate stigma). Disordered eating cognitions and psychological inflexibility were both associated with greater psychological distress and lower help-seeking stigma tolerance. Similarly, psychological distress influenced help-seeking stigma tolerance through its effect on psychological inflexibility and disordered eating cognitions. |
| McAndrew & Menna, 2014 | Cross-sectional study | n=198 | Undergraduate females taking a psychology course at a Canadian university; participants were randomly assigned to read a vignette from their perspective or from the perspective of a hypothetical female student named “Lauren” | 20.08 (1.58) [18-25] | EDE-Q (eating pathology); EAT-26 (symptoms); Barriers to Adolescent Seeking Help Scale—Brief Version (BASH-B); Attitudes Towards Seeking Professional Help Questionnaire—Short Form (ATSPPH); participant’s perceptions of the behaviours of the person in the vignette (themselves or “Lauren”) | Pearson correlations; Chi Square tests; multiple regression analyses; multinomial logistic regressions; independent samples *t*-tests | Relative to women that read the vignette about “Lauren” (n=100), women who read the vignette about themselves (n=98) were more likely to 1) attribute their own behavior to a general mental health issue (not an ED), 2) believe that they were able to cope with their problem alone (without help), and 3) believe that they did not need to seek help for their food-related behaviors (despite perceiving fewer barriers to doing so). For women who read the vignette about themselves, there was a stronger positive association between perceived severity of the issue and the belief that the character should seek help, and a stronger negative association between perceived ability to cope with the issue alone and belief that the character should seek help. |
| McLean et al., 2019 | Cross-sectional study | n=200 | Individuals who visited the Reach Out and Recover website | 44% of the sample was 20-29, 21% was 18-19  [18-60] | Treatment-seeking status; frequency of engagement with disordered eating behaviors; Appearance dissatisfaction; dysfunctional, rigid, or inflexible thoughts about eating, food, body image; impact of ED symptoms on mental health, relationships, well-being; motivation, confidence, stigma, and ambivalence about change | Spearman correlation coefficients (e.g., relationships among treatment-seeking, perceptions of treatment, and ED symptoms and their impact) | 86% of participants experiencing a range of ED symptoms were not receiving treatment (n=172), but of these, 82.6% indicated that they planned to get treatment. Intention to seek treatment was positively associated with motivation to change, confidence to achieve change, greater frequency of binge eating, and greater recognition of the impact of ED symptoms on relationships and well- being but not with stigma or ambivalence. Participants' choice to download the health-professional report was also used as an indicator of treatment-seeking intention. |
| Meyer, 2005 | Cross-sectional study | n=294 | Undergraduate women enrolled in an introductory psychology course at a large midwestern university | 19.33 (1.99) [17-32] | Questionnaire for Eating Disorder Diagnoses (Q-EDD); Thoughts About Psychotherapy Survey (TAPS); Beliefs About Attractiveness Scale–Revised (BAA-R); Defense Style Questionnaire–40 (DSQ-40; Demographics; Eating Concerns; treatment history or intention to seek out treatment | Multivariate analysis of variance (MANOVA) was conducted to compare the participant groups on dependent variable measures. | 74 reported some degree of ED symptomatology’; 2 were seeing a therapist for their eating concerns. 32 (11%) met ED criteria and 5 were currently seeing a therapist for their ED. In terms of whether each participant thought they needed counselling for their ED symptoms, 56% (n=18) of the ED participants and 39% (n=29) of the symptomatic participants thought they did, while 44% (n = 14) of ED participants and 61% (n = 45) of symptomatic participants did not think they needed counselling. Across both groups, females who did not think they needed treatment engaged in higher levels of immature psychological defenses (e.g., denial) and were less likely to endorse sociocultural norms (i.e., less internalization of societal beauty ideals) than those who though they needed treatment. |
| Musolino et al., 2016 | Mixed Methods | n=21 | South Australian women who had not received treatment for disordered eating (formal diagnosis did not matter) | [19-52] | EDE-Q; eating and exercise habits; whether considered their activities ‘a problem’; what cultural ‘norms’ supported their eating and exercise; whether they had considered seeking help; 8 weeks-worth of participants’ daily eating and exercise habits that supported their disordered eating behaviours | Ethnographic and grounded theory principles guided the research. Thematic analysis was used to analyze interview, observation, and field note data. | 19 met ED criteria and had not previously sought professional help or received diagnosis. Reported not seeking help because they perceived their disordered eating as ‘safe’ (maintaining routines, eating ‘safe’ foods). Participants were scared to gain weight, did not see their routines as problematic, and felt recovery was not possible. Participants noted how being surrounded by society’s emphasis on thinness and dieting contradicted any motivation to seek help or recover. Many reported being able to hide their practices within normative cultural ideals around food and body (e.g., food allergies, intolerances), as their desire to constantly work on their bodies through over-exercise was accepted. |
| Neyland & Bardone-Cone, 2019 | Cross-sectional study | n=43 | A sub-sample of Latina women (from a larger study sample) with a history of BED and/or BN | 20.58 (2.12) [18-25] | ED Diagnostic Scale (EDDS) (lifetime and current ED diagnosis); treatment history, experiences, perceived helpfulness; barriers to treatment; Stephenson Multigroup Acculturation Scale (SMAS) (factors associated with treatment experiences); ATSPPH - Stigma Tolerance subscale | One-way ANOVAs were conducted to determine statistically significant differences between variables. | 65% (n=28) sought treatment. Most frequently seen healthcare professionals: psychologist/ therapist for individual therapy and nutritionist/dietician (68%). Participants endorsed “other” treatment providers as being the most helpful (e.g., “specialist on EDs”, social worker, yoga teacher, and an endocrinologist). Psychologist and therapist-led group therapy was rated as most helpful, while treatment by a physician was rated as least helpful. Participants endorsed cost of treatment, ED stigma/ shame, and mental health shame as the most influential barriers to seeking or receiving treatment. |
| Plateau et al., 2017 | Qualitative study | n=13 | Female athletes currently receiving treatment for an ED | 23.95 (8.04) | Experiences and perspectives on seeking and receiving ED treatment | Thematic analysis of semi-structured interviews | Challenges to treatment-seeking included: lack of ED literacy among athletes and their coaches, difficulties accepting ED, practical barriers to treatment seeking, perceived availability of emotional support from family members, coaches, friends, and partners. Athletes felt out of place and uncomfortable in the treatment context e.g, difficulty maintaining athletic identity, perceived lack of relevance of therapy. |
| Potterton et al., 2020 | Qualitative Study | n=14 | British emerging adults with an ED in, or had previously received, outpatient or day treatment (subset from FREED-Up study) | 20.9 (2.0)  [18-25] | Attitudes towards ED symptoms, and their implications for help-seeking | Thematic analysis consistent with the critical realist framework; coding was informed by existing theory and literature on emerging adulthood and ED-related help-seeking | Symptom egosyntonicity during early stages led participants to not reach out for help; parents usually sought help on their behalf. For some, a life change was a help-seeking deterrent (e.g., “fresh start”). Vague/generic comments from others decreased help-seeking; concern and support (scheduling, attending) from family and friends facilitated it. Help-seeking was also avoided due to belief that they did not fit into the classic definition of ED (e.g., not thin enough). Fear of rejection, shame, and embarrassment led participants to want to handle it alone and not seek help. Some participants chose to seek help without their parents knowing and did not go to GP or teachers in case that information was shared with parents. Some chose indirect ways for help-seeking because they were “less embarrassing”, such as written communication, generic mention about stress, and discussing physical health. |
| Prouty et al., 2002 | Cross-sectional study | n=578 | A random sample of female undergraduate and graduate students enrolled at a large, public, mid-Atlantic, rural university | [18-22+] | EAT-26; who participants would go to for help for weight or eating habits or eating problem; participants’ first and second choices for support if they chose to go to therapy | Chi-square tests of independence | Participants reported they would most likely go to their friend if they were concerned about their weight or eating habits; those who scored negative for an ED chose physicians as their second choice, while those who scored positive for an ED selected dieticians as their second choice. Participants reported choosing individual therapy first if they needed help with an eating problem. In second place, however, women who scored negative for an ED chose a dietician, while women who did score positive chose group therapy. Both groups chose a close friend as the person to support their work in therapy; a significant other, parents, and siblings were also chosen. |
| Romano & Lipson, 2019 | Cross-Sectional Study | n=3201 | American university students from the general population (sub-study of the Healthy Bodies Study) | 24.96 (13.86) | EDE-Q (restraint); ED stigma; Patient Health Questionnaire (PHQ-4) (negative affect); perceived ED treatment need | Structural equation mixture modeling to: 1) examine how different restraint symptoms cluster among women and men; 2) determine if gender differences exist for ED stigma, negative affect, and  perceived ED | Compared to women who show higher restraint, more associations between variables of interest were found among women with lower restraint: increased ED stigma and increased negative affect were associated with increased perceived need for treatment. The opposite was true for men: among men with high restraint, elevated ED stigma and negative affect were associated with increased agreement of perceived need for help. |
| Rother & Buckroyd, 2004 | Qualitative study | 6 | Adults from the UK who had recovered from their ED recruited from a voluntary sector agency | [18-28] | General information about participants and their circumstances; timing of the onset of their eating disorder and how it had manifested itself; when they first felt they had a problem and needed help; what they felt about any help they might have received at that time and on a continuing basis; what they would have liked at that time; where they would have liked that help to be based | Thematic Qualitative analysis | Ambivalence to reach out for help was rooted in desire to keep ED and habits hidden. Primary care professionals were ill-informed, unable to identify ED warning signs, and unsure of needs of adolescents with EDs (e.g., when mothers approached GPs about their child, GPs usually had negative responses— “it’s a teenage thing”). One participant found that their GP’s disclosure about having experienced BN helped gain their trust. In-patient treatment did not address the underlying reasons for the ED (e.g., anxiety, depression). Media was identified as a destructive force. All 6 participants approached this agency as their last hope; found that the main therapist at the volunteer sector agency they were recruited from understood them, and they shifted the focus from food to self-esteem, problem-solving, etc. Felt schools should be more involved in educating about the destructive nature of EDs, requested more information that was easily and anonymously attainable be made available, and recommended more maternal ED education, since their mothers influenced initial ED thoughts. |
| Schwitzer et al., 2008 | Cross-sectional study | n=122 | Female college students who either (1) participated in a National Eating Disorders Awareness week activity or (2) were presenting with eating concerns at the campus mental health centre that week | 21.92 (5.73) | EAT-26; Standardized Health Behavior Questions (frequency of engaging in ED behaviours in past 3 months, height, weight, suicidal ideation, help-seeking) | Descriptive and comparative analysis of screening instruments to compare primary symptoms, associated features, and help-seeking | This study identified 22 women with clinically significant eating concerns who sought no treatment and 63 women with no clinically significant eating concerns. No participants in the eating-concerns group reported seeking treatment from a medical doctor, psychiatrist, psychologist, or counselor. Alternatively, about one-fourth of the women reported seeking assistance from adjunct services, including either a nutritionist or a support group. Of the 34 women who presented for first-time intake appointments at the mental health centre on campus, none of them presented eating-related problems as their reason for seeking treatment. |
| Schwitzer et al., 2001 | Cross-sectional study | n=130 | Female college students who used a multidisciplinary eating disorder intervention program from 1990-1995, whose records provided sufficient data to address study outcomes | Mode = 19 [17-24] | Narrative descriptions of presenting concerns; General psychosocial problem checklist; Health and Lifestyle Assessment (self-report, categorical, and chronological information about current or recent DE, treatment, history, family relationships); EDI; body dissatisfaction | Not reported | Participants self-referred (39%, n=52), were referred by a friend (34%, n=45) or relative (7%, n=9), by members of the ED program, by health center physicians (5%, n=7), by academic advisors (4%, n=5), or by residence life staff (4%, n=5). 38% (n=50) reported having tried individual counselling for eating concerns at least once, and 11% (n=15) reported having been hospitalized for ED before coming to college. |
| Smalec & Klingle, 2000 | Cross-sectional study | n=44 | Participants who self-described as bulimic and were currently receiving help for their ED were recruited from Overeater Anonymous meetings (52%), Internet news group (30%), and through word of mouth (18%) | 24 [15-38] | Demographic information, ED-specific questions (e.g., duration of disorder, current treatment); questions regarding their help-seeking beliefs; items that assess perceived threat and efficacy on message acceptance and rejection; factors that influence help-seeking behaviour (e.g., relational closeness) | Descriptive statistics were used to describe the sample’s demographic information and ED-specific questions. ANOVAs determined effects and interactions between and within the variables of threat and efficacy. | 64% of participants reported that interpersonal communication influenced them to seek help.  It was found that individuals with bulimia with high levels of efficacy (belief that they can seek and receive help) are more likely to seek help.  When threats that one’s ED symptoms are dangerous or harmful are present, these threats work to motivate individuals with bulimia with high efficacy only—not those with low efficacy. |
| Tavolacci et al., 2020 | Cross-sectional study | n= 1493 | Students from a French university | 20.1 (1.9) [  <26 years] | SCOFF (for ED screening); DASS-21; asked: “if had seen general practitioner in last 12 months and if so why?”, "have you foregone seeing a doctor", “resources for help for emotional problems?” | Chi-square tests were used for categorical data, and *t-*tests and descriptive statistics were used for continuous variables. | The prevalence of likely ED cases was 24.8%. Among those with EDs, friends and family were the main resources for help-seeking in emotional stress situations. Compared to students without EDs, students with EDs consulted their GP more often for stress or anxiety, had a greater risk of renouncing treatment, especially related to a fear of seeing a GP; also wanted to solve problem on own. In emotionally stressful situations, students with restrictive EDs sought help from a psychologist or a specialist practitioner. |
| Thapliyal et al., 2020 | Qualitative study - Retrospective* | n=8 | Australian men who had been diagnosed, sought help, and were treated for an ED | 26.14 [20-33]  *age of onset was younger | ED experiences; help-seeking behaviours; treatment experiences | Thematic analysis | Help-seeking barriers: 1) Minimization of the ED and help-seeking as a man (did not conceive symptoms as problematic nor an ED, thought they were just “eating healthy” (“safe”), lack of ED awareness, ED=coping mechanism, provided self-confidence or sense of achievement, part of their identity; 2) Idea that men do not suffer from EDs (pattern of stigmatization that this is the problem of young females or gay men, masculinity was challenged); 3) Negotiating treatment (being misdiagnosed, therapist's lack of understanding of male EDs, lack of treatment facilities and supports tailored to their needs); 4) Gender bias (sense of isolation being the only male, treatment is not tailored to mens' needs). |
| Tillman et al., 2015 | Longitudinal study | n=136 | Undergraduate and graduate students who attended campus- wide programming for NEDAwareness Week | Not reported. It can be assumed that participants were emerging adults given that the sample was comprised of undergraduate and graduate students. | Knowledge of Campus Resources Scale; EAT-26; Eating Disorder Help-Seeking Behaviours Inventory: Self and Friend Versions; Objectified Body Consciousness Scale (Body Shame subscale) | Single paired sample *t-*test to evaluate degree to which students with high levels of eating concerns would seek help for themselves and friends. Multiple regression analysis was used to evaluate how body image and eating concerns predicted help-seeking behaviour. | 14% of the sample reported high levels of eating concerns. Among students who attended this programming, 83% endorsed that they would seek help for their friends with an ED but only 35.3% endorsed that they would seek help for themselves if they were experiencing an ED. Specifically, although those with low and high levels of eating concerns were both significantly more likely to seek help for a friend with an ED after attending the programming, only those with low levels of eating concerns were significantly more likely to seek help for themselves after the programming. Out of those with high levels of eating concerns, only 21% endorsed that they would seek help for themselves. |
| Tillman & Sell, 2013 | Cross-sectional study | n=108 | Students at a mid-sized American university | [18-24] | Attitudes Toward Seeking Professional Psychological Help Scale-Short Form; Knowledge of Campus Resources Scale; Perceived Knowledge of Eating Disorders Scale; help-seeking behaviors for self and for a friend experiencing an ED | Maximum likelihood factor analysis (e.g., Perceived Knowledge of Eating Disorders Scale); paired sample *t*-test; multiple regression analyses | Participants were significantly more likely to seek help for a friend with an ED than to seek help for themselves if they were experiencing an ED. Only the knowledge of EDs significantly predicted whether a student would be willing to seek help for a friend with a general psychological disorder. No factors (i.e., sex, year in college, knowledge of EDs, and knowledge of available resources) predicted willingness to seek help for friends with an ED. |
| Valente et al., 2020 | Mixed Methods | n=185 (Quan); n=10 from Quan (Qual) | Quan: Individuals who self-diagnose as having orthorexia nervosa (ON) or post content related to ON  Qual: purposeful subsample from participants in quan phase | Median=24 [16-55] | Quan: opinion about ON, personal history, experiences with ON (if applicable)  Quan: experiences and development of ON (“onset,” “progression” and “help seeking.”) | Quan: descriptive statistics (presence and intensity of different drivers and symptoms); Chi-square, t-tests and analysis of variance tests (ANOVAs) were used to compare subgroups.  Qual: Complementary mixed methods approach was used to generate themes. | 77% of respondents who self-diagnose with ON (SD-ON) considered treatment at some point. For those who realized that they had a problem (negative impact on life), this was usually sparked by difficulties that were not easily observed by others (e.g., anxiety, pain, or discomfort due to malnutrition) and feelings of isolation when relationships suffered. One barrier for recovery was that ON was not noticed by loved ones until severe weight loss or major health problems occurred. Respondents reported another barrier to recovery as “the ineffective healthcare system where health professionals lack knowledge about ON and disordered eating”. |
| Wacker, 2018 | Qualitative study | n=15 | Women with subclinical eating disorders without a clinical diagnosis or a treatment history | 21 (1.62)  [18-25] | EAT-26 (to determine subclinical ED status); demographic questionnaire; interview regarding treatment-seeking and supportive relationships | Feminist-informed constructivist grounded theory; open, axial, and theoretical codes were developed. | **Barriers** to seeking treatment: 1) Personal reasons (not ready, not wanting help, benefits of symptoms outweighed perceived cost of help-seeking, fear of weight gain, can control alone, lack of resources to access treatment); 2) Lack of openness with support system (not wanting others to worry or feel burdened, fear that their support person will blame themselves, feel bad or angry, push treatment); 3) Misperceptions of subclinical symptoms (symptoms not extreme enough for treatment, EDs are only AN or BN, normalizing symptoms, denial); 4) Stigma (shame and embarrassment around symptoms, fear of being judged, mental health stigma). **Facilitators** for treatment included: 1) Relational empowerment (values relationship with support person, support person noticed and challenges symptoms, honesty/openness); 2) Utilization of shared experiences/self-disclosure (shared experience of MH struggles are helpful 🡪 openness about symptoms, decreased shame); 3) Understanding severity of issue (treating subclinical symptoms with seriousness, avoid normalizing body dissatisfaction); 4) Importance of emotional and tangible support (support person doesn’t try to “fix”, offering humor, solutions, distractions, empathy, love, trust, care. |
| *Participants are retrospectively reflecting on their experiences when they first sought treatment. Their age at the time of the study does not reflect the age related to their responses about help-seeking. | | | | | | | |

**Table 3.** Eligible studies with samples of individuals including a mixed population of children/adolescents and emerging adults (those aged younger and older than 18) (n=12)

| **Reference** | **Study Type** | **Sample Size** | **Sample Description** | **Mean Age (SD) [Range]** | **Help-Seeking related Outcomes Reported** | **Type of Analysis** | **Help-Seeking related Findings** |
| --- | --- | --- | --- | --- | --- | --- | --- |
| Aardoom et al., 2014 | Cross-sectional study | n=311 | Individuals who indicated having eating problems who were engaging with the Proud2Bme website (empowers individuals with ED symptoms and promotes positive body image and a healthy lifestyle) | 20.2 (5.0)  [13-40] | EDE-Q; experience of empowering processes and outcomes because of visiting/using Proud2Bme; Dutch Empowerment Questionnaire (NEV) | *T*-tests and ANOVAs conducted to examine whether experiences of empowering processes and outcomes differed according to self-reported ED diagnosis, treatment status, frequency of web visits, etc. | To a small degree, participants experienced increased help-seeking behavior, increased optimism, and control over the future, and increased confidence in treatment and the relationship with the therapist because of engaging with the website. 53% of participants were using the website to 'find help'. Higher levels of ED psychopathology were associated with increased help-seeking behaviour but also lower self-esteem. |
| Dooley-Hash et al., 2013 | Cross-sectional study | n=1920 | Patients from the Project Uconnect study who presented to the emergency department at a Michigan medical centre with an ED from Oct 2010 to Sept 2011 | 17.5 (2.0)  [14-20] | BMI; SCOFF (ED screening questions); frequency of emergency department visits by patients during 12 months prior to their index visit; patients' primary reason for their emergency visit | Bivariate analyses and Chi-squared tests; negative binomial regression model | Emergency department patients who screened positive for EDs were significantly more likely to have visited the emergency department before and, on average, utilized the emergency department at a rate 1.6 times higher than patients who screened negative for EDs. Among these patients, the most common chief complaints were abdominal pain and other gastrointestinal-related problems. |
| Fitzsimmons-Craft, Balantekin, et al., 2020 | Cross-sectional study | n= 343,072; of these, n=16,396 (4.8%) completed help-seeking intention question;  n=2,765 completed 2-month follow-up | Individuals who completed NEDA’s online screen over an 18-month-period (Feb 2018-Aug 2019) who screened positive or as high risk for an ED | 86.5% of sample was between the ages of 13 and 34 | All respondents were provided the Stanford-Washington University Eating Disorder Screen (SWED). Those who screened positive or as high risk for an ED, were asked about their intention to seek professional help and were asked if they would opt-in for a 2-month follow-up survey. Those who agreed were asked about their help-seeking behaviours and reasons for seeking treatment at 2-month follow-up. | Descriptive statistics; chi-square tests were used to compare ED diagnostic/risk and demographic groups on help-seeking intentions. | Of those who completed the help-seeking question (n=16,396), 10.3% reported they would definitely seek help, 23.4% probably, 40.8% probably not, and 25.5% not. Groups who indicated that they definitely/probably would seek help were those with clinical/subclinical BED vs other diagnoses and females and individuals of other genders vs males. Of all age groups, those ≤17 were least likely to. The most preferred type of help was to make an appointment with a mental health professional (49.5%) or medical doctor (30.1%), in a respondent pool of 6,264. Among those who completed 2-month follow-up, their primary reasons for seeking treatment: emotional distress (25.2%), concerns with eating (17.3%), weight (16.3%), or health (10.8%), encouragement of friends (8.2%), referral from the NEDA screen (7.5%), and initiated by parents (7.3%). Of the 2,765 respondents who completed the two-month follow-up survey, 8.9% reported being in treatment when they took the screen, 15.5% subsequently initiated treatment, and 75.5% did not initiate or were not already in treatment. Of follow-up respondents who endorsed seeking treatment (n = 694), 67.4% made appointment with a mental health professional, 47% made appointment with a medical doctor, 8.5% attended a support group, 5.0% contacted the NEDA Helpline for treatment referrals, and 4.2% signed up for the recommended online/mobile program. |
| Fitzsimmons-Craft, Krauss, et al., 2020 | Cross-sectional study | n=405 | English-speaking American youth who demonstrated an interest in and/or followed pro-ED social media accounts | Mean not reported  [15-17]  (51% of the sample was children/ adolescents); mean not reported  [18-25]  (49% of the sample was emerging adults) | Use of pro-ED social media over the past month; Stanford-Washington Eating Disorder Screen (SWED) (ED risk, type of ED); Eating Disorders Quality of Life instrument (EDQOL); Patient Health Questionnaire (PHQ-9) (psychological comorbidities); treatment history or barriers to treatment; opinions of leveraging social media to recruit participants for ED studies and to connect people to ED treatment | Descriptive statistics; Pearson Chi-square tests; Mann-Whitney U tests | 14% of adolescents and young adults with clinical/subclinical EDs, had received treatment.  Reasons for not seeking treatment: believing the problem was not serious enough (>80% for each age group), one should help themselves (>70% for each age group), worrying about being labeled/ judged (>50% for each group). Young adults were more likely than adolescents to express concerns about stigma (50% vs 35%). Adolescents were more likely than young adults to believe that they did not have a problem with an ED (32% vs 20%), that an ED is not a psychological problem (27% vs 17%), and to fear being separated from their family (30% vs 19%). 84% of participants indicated an interest in trying a mobile mental health program with a human component. |
| Flatt et al., 2020 | Cross-sectional study | n=23,920 | Individuals who fully completed NEDA's online screening tool during National Eating Disorders Awareness Week in 2018 and self-identified as athletes or non-athletes | 38.5% (ages 13-17)  44.1% (ages 18-24)  17.4% (25+) | Stanford Washington-Eating Disorders Screen (SWED; assesses disordered eating behaviours; probable ED diagnosis/risk); treatment history; intent to seek treatment | Descriptive statistics; adjusted permutation *t*-tests; logistic regression; exploratory chi-square test | Of 86% of respondents who met criteria for an ED/subthreshold ED (n= 20,727), 2.5% were in treatment and 75% reported that they would not seek treatment. No significant differences between athletes and non-athletes emerged on treatment history or intention to seek treatment post-screen. Respondents who indicated they were likely to seek treatment reported they would seek help from a mental health professional or a medical doctor. |
| Griffiths et al., 2014 | Cross-sectional study | n=317 | International sample of individuals with a currently diagnosed ED | 24.68 (7.18) [14-54] | Stigmatizing attitudes and beliefs; Self-Stigma of Seeking Help scale (SSOSH; self-stigma associated with seeking psychological help); Depression Anxiety Stress Scales (DASS-21); Self-Esteem Scale (SES; global self-esteem); EDE-Q | Significant univariate ANOVAs were followed up by contrast analyses  Spearman correlational analysis;  Exploratory factor analysis and multiple regression were also used. | Most participants were currently receiving treatment for their eating disorder (57.1%), 32.8% were not currently receiving treatment but had received treatment in the past, 7.3% had never received treatment. Participants who experienced more frequent stigmatization for having an ED were more likely to hold self-stigma for seeking psychological help. |
| Kästner et al., 2021 | Qualitative Study | n=4 adolescents,  6 adults | Female patients with AN aged 14 or older new to treatment (from the larger FABIANA-study) | Adolescents: 15.75 (1.50) [14-17]  Adults: 23.33 (7.47) [20-38] | Weight; height; date of treatment initiation; semi-structured interviews about facilitators and barriers for treatment initiation | Categories (and their frequencies) were informed by codes following the Grounded Theory approach. | Identified facilitators to treatment initiation: experiencing somatic symptoms (brittle nails, pericardial effusion); symptom exacerbation and occurrence of a “breaking point”; not living or being alone; early recognition, concern; continuity and persistence in making AN-symptoms the subject of discussion by family and friends; positive relationships with others with open/calm exchange, support/understanding; exposure to positive role models who went through treatment; supportive reminders for appointments, encouragement to seek/continue with treatment; knowledge about EDs, consequences, management among referring GPs; formal detection of /communication about an ED; clinical recommendations/referrals that were binding, timely, clear, and concrete; regular control examinations or continuity of treatment with GPs. Participants identified the following barriers: lack of understanding of AN and need for treatment among friends and family; underestimation of severity and stigmatizing beliefs by close others; close exposure to others with existing ED; long wait times and limited availability of treatment; healthcare providers failing to recognize ED due to the patient coming in for something else; previous experience of normalizing, trivializing, or neglecting ED pathology by a healthcare provider; stigma surrounding AN and therapy; comparison to social media. |
| Moessner et al., 2016 | Open trial | n=453 | Participants using ProYouth; open to all but encouraged for those at-risk of EDs or with slight symptoms who were not already in treatment | 15.7 (4.8)  [12-56]  *84.3% were high school students, 7.1% were university students | After screening, participants were asked 3 months after registration about: whether they have accessed professional help for eating or body image concerns in past 3 months; if not, participants were asked if they intended to seek help; if not, participants were asked for the reasons (barriers) why they would not access conventional healthcare | Group differences investigated using chi-square methods for categorical data and t-tests for metric variables. Differences in utilization of program tested using Mann-Whitney U-tests. | At 3 months, 43 (9.5%) took up treatment during their first 3 months using ProYouth, 32 (7.8%) intended to start treatment, and 163 (43.1%) of the remaining reported that they would seek professional help if they had a need for it. 50% of potential help-seekers reported ProYouth changed their attitude towards help-seeking. Mental health literacy (problem not serious enough to utilize professional help) and shame/stigma ("I would not want anybody to know about it") were most frequently mentioned barriers to help-seeking. Those who reported having sought professional help or intentions to do so (n=195): older, female, had greater impairment at registration, acknowledge more that they need help, held more positive expectations about the efficacy of professional support, and used ProYouth more frequently than those who did not intend to seek treatment. |
| Neubauer et al., 2014 | Cross-sectional study - Retrospective* | n=140 | German patients receiving treatment for AN, who had an early onset (<14 years, n=40), intermediate onset (15-18 years, n=53), or late onset (19> years, n=47) | Age of onset: 17.51 (5.81)  Current age: 22.34 (6.79) | Structured Clinical Interview for DSM-IV (SCID; ED diagnosis, onset, and duration of untreated illness); history regarding first treatment; EDE-Q (eating pathology); Patient Health Questionnaire (PHQ-9; depression) | Binary logistic regression to assess differences between onset groups and person who first diagnosed them, first treatment facility and setting, participants’ motivation. Negative binomial regression was used to assess differences in duration of untreated illness. | The late onset group was more likely to be internally motivated (68.1%) to seek help than the intermediate onset group (50.9%) and early onset group (37.5%). Furthermore, the early onset group was more likely to be informed about first treatment facility by their social network (e.g., parent or friend) (47.5%) than the intermediate (35.8%) and late (19.1%) groups; oppositely, the late onset group was more likely to receive information about the first treatment facility from a physical health professional (61.7%) than the intermediate (52.8%) and early (40.0%) onset groups. |
| Raisanen & Hunt, 2014 | Qualitative study - Retrospective* | n=10 | Young males with an ED living in the UK who were interviewed as part of a larger study to inform an online patient information resource | Age at interview: 20.3 [17-25]  Age at diagnosis: 17.25 [14-22] | ED experiences; barriers to accessing primary care; experiences of health professionals’ responses to their initial presentations of ED signs and symptoms | Qualitative interpretative approach, using inductive close reading and constant comparison of qualitative interview data | Participants’ initial inability to identify ED symptoms was a result of their gendered understanding of EDs (e.g., did not consider it as an option, thought it was a young teenage girl issue). Friends, family, teachers, and health professionals were reported as also being unable to recognize their ED due to their gender. Men delayed help-seeking because they had become entrenched in routines and felt hesitant to change. Once the problem was no longer private, impeded social life or well-being, or they hit a crisis point (e.g., hospital admission) was when they recognized things were not right. Men held fears (or past experiences) of not being taken seriously by healthcare providers. Participants feared judgment, hurting others’ feelings, not wanting to ‘burden’ services, and forced intervention. Men did not know where to seek help and noted that there was a lack of male-targeted ED resources. Mothers took them to their first appointment once they decided to seek help. Participants’ first contact with formal services was usually their GP. They reported having mixed experiences with professionals (e.g., positive, repeated appointments before being referred, misdiagnosed). |
| Reid et al., 2008 | Qualitative study | n=20 | Young individuals receiving outpatient treatment for AN or BN | [7-41] | Participants’ perceptions of how their ED had affected them; treatment experiences; perceptions of the strengths and weaknesses of the outpatient service | Thematic analysis using a 6-phase semantic approach | Participants reported that their ED initially provided them with a sense of control until it became too much to cope with, leading to suicidal thoughts and desperation, which influenced them to seek treatment. However, participants were concerned that receiving treatment would make them lose control (e.g., gain weight). An important factor that allows participants to feel comfortable to seek help is if were guaranteed some level of control still—a balance of autonomy and direction. This can be achieved if participants who want to be involved in treatment decisions are included in these conversations. Long referral times were another deterrent in the process of help-seeking. |
| Richardson et al., 2020 | Cross-sectional study | NEDIC was contacted 609 times (72.1% individuals affected, 20.4% caregivers) | NEDIC service utilization data | 2% of sample were 11-14 years, 29% were 15-19 years, 25% 20-25 years, 31% were 26+, and 13% were unknown | Service utilization statistics during the initial pandemic period (March 1–April 30, 2020) and the corresponding time periods in previous years (March 1–April 30, 2018 and 2019); self-reported diagnosis; reason for contacting NEDIC; rates of symptoms; behaviours present | Non-parametric chi-square tests used to compare frequencies of help-seeking behaviors between years among groups. Chi-square tests to assess differences in symptom frequency between years. Thematic analysis of chats. | NEDIC saw a large increase in help-seeking rates during 2020 pandemic months (n=609), compared to the corresponding period in 2018. Number of total contacts significantly increased from 2018 (n=394) to 2019 (n=572) and 2018 to 2020 (n=609) (X2(3) = 50.34, p < .001). There were higher rates of ED symptoms, anxiety, and depression in 2020 compared to previous years. Thematic analysis of instant chats from the pandemic year revealed four emerging themes: 1) lack of access to treatment, 2) worsening of symptoms, 3) feeling out of control, and 4) need for support. |
| *Participants are retrospectively reflecting on their experiences when they first sought treatment. Their age at the time of the study does not reflect the age related to their responses about help-seeking. | | | | | | | |
